# The role of AI-enhanced fast delivery services in strengthening customer retention and loyalty in competitive markets

**DOI:** 10.3389/frai.2025.1612772

**Published:** 2025-10-13

**Authors:** Apoorva Kasoju, Tejavardhana Vishwakarma, Abhinaya Kasoju

**Affiliations:** ^1^Amazon, Lynnwood, WA, United States; ^2^Metro Markets, Berlin, Germany

**Keywords:** AI-enhanced delivery, predictive analytics, reinforcement learning, customer personalization, last-mile delivery, operational efficiency

## Abstract

This research presents an AI-enhanced framework to optimize last-mile delivery systems by integrating predictive analytics, Reinforcement Learning (RL), and customer personalization. The predictive analytics component utilized XGBoost and Random Forest models to forecast delivery times. Random Forest achieved better performance, with a Root Mean Square Error of 1.52 and an R-squared value of 0.56. RL-based route optimization improved operational efficiency by reducing the average delivery time from 31.2 to 25.4 min, increasing timely deliveries from 78\% to 92\%, and reducing idle time by 15\%. Customer personalization, driven by sentiment analysis and clustering, increased positive sentiment from 68\% to 80\%. It also improved Net Promoter Scores from 68 to 85 and increased customer retention from 74\% to 89\%. The proposed framework addresses the challenges of last-mile delivery by combining data-driven predictions, adaptive routing, and personalized customer strategies. Future work will explore real-world implementation using real-time traffic data and advanced personalization techniques to improve adaptability and scalability.

## 1 Introduction

The rise of e-commerce and on-demand services has fundamentally transformed consumer expectations about delivery speed and quality (Zhou et al., 2025). Today, customers prioritize fast, reliable, and personalized delivery services, often making purchasing decisions based on the promise of timely delivery (Zhou et al., 2025). In response, businesses are increasingly adopting AI-driven technologies to enhance their last-mile delivery capabilities (Islam et al., 2024). These technologies aim to optimize route planning, predict delivery times, and personalize the delivery experience (Muthukalyani, 2024). This caters to the ever-growing demands of modern consumers. AI-powered delivery systems improve operational efficiency and impact customer satisfaction and loyalty (P. Singh and Singh, 2024). Efficient delivery is critical for maintaining customers in competitive markets, as delays or inaccuracies can lead to negative experiences and customer churn (Bahashwan, 2025). By using machine learning, predictive analytics, and real-time˜ data, businesses can address these challenges (Martínez-Troncoso and Solis, 2025). This fosters a smooth delivery process and improves relationships with customers. Despite advances, existing delivery systems face challenges in scalability and adaptability to dynamic market conditions (Johnson et al., 2024). In addition, they struggle with the integration of customer feedback into delivery processes (Ajike et al., 2025). These gaps highlight the need for an improved framework that combines operational excellence with customer-centric features. This research is driven by the chance to investigate how AI-powered delivery systems can close existing gaps. Establish new standards for customer retention and loyalty within competitive markets.

This research focuses on optimizing AI-driven delivery systems to boost customer retention and loyalty in competitive markets. It also addresses operational inefficiencies and customer satisfaction issues. Existing solutions largely rely on standalone AI tools for specific tasks. These tasks include route optimization, demand forecasting, or customer feedback analysis. Although these systems have shown promise, they often operate in silos and do not provide an integrated view of the delivery ecosystem (Ajike et al., 2025). Many current implementations lack personalization features and robust feedback loops. This limitation affects their ability to adapt to individual customer preferences and evolving market demands. It is necessary to build comprehensive frameworks that holistically address both operational and customer-centric goals (Bennett, 2024).

We propose a comprehensive AI-enhanced delivery framework. This framework integrates predictive analytics, route optimization, and personalized customer engagement features. Unlike existing systems, our approach emphasizes the seamless integration of operational efficiency and customer-centric design. This is achieved by using real-time data and adaptive learning models. This unified framework not only improves delivery speed and accuracy, but also enhances customer satisfaction by offering personalized services. Consequently, it cultivates enhanced retention and loyalty. By addressing the limitations of current systems, our solution provides a more scalable and adaptable approach to last-mile delivery challenges.

To address the research problem, the following research questions are formulated:

1. What are the key challenges faced by current delivery systems in ensuring customer retention and loyalty?
2. How can AI technologies be used to optimize delivery operations and enhance customer satisfaction?
3. What are the measurable impacts of AI-driven delivery systems on customer retention and operational efficiency?
4. How does the proposed framework compare with existing solutions in terms of scalability and adaptability?

This research is significant because it addresses a critical gap in the intersection of operational efficiency and customer experience in last-mile delivery services. Using AI technologies, this study aims to provide a scalable and adaptable solution that meets the demands of modern consumers. The findings of this research can help businesses achieve a competitive edge by improving customer retention and loyalty, which are key in highly competitive markets. Furthermore, this study contributes to the academic and industry discourse on AI applications in logistics and supply chain management. By offering a holistic view of AI-enhanced delivery systems, this research paves the way for future advancements, bridging the gap between theoretical innovation and practical implementation.

The objectives of this study are four-fold. First, it identifies the current challenges and limitations faced by AI-based delivery systems in competitive markets. Second, it presents an integrated framework that combines predictive analytics, reinforcement-based route optimization, and customer personalization. Third, it evaluates the framework using real-world data sets to assess improvements in customer satisfaction, delivery accuracy, and operational efficiency. Fourth, it offers practical recommendations for implementation in commercial contexts. These objectives are addressed in the methodology (Section 3), analyzed through experimental results (Section 5), and further discussed in terms of comparative performance (Section 6).

The remainder of this paper is organized as follows. The next section reviews the related literature on AI-enhanced delivery systems and customer retention strategies. This is followed by a detailed discussion of the proposed AI-powered delivery framework and its components. The methodology and data sources used in this study are then described, leading to the results and analysis. The discussion section interprets the findings and their implications, and the paper concludes with a summary of the study and directions for future research.

## 2 Literature review

The use of AI to enhance customer experiences, operational efficiency, and business decision-making is gaining traction across industries. This section reviews key studies, categorized into AI in customer engagement, CRM, supply chain management, and industry-specific innovations.

Kumar et al. investigated AI-powered marketing strategies, highlighting their impact on improving customer engagement and decision-making efficiency (Kumar et al., 2024). Their study employed real world marketing datasets to demonstrate AI’s ability to segment customers and drive targeted campaigns. Prem and D focused on hyper-personalization in FMCG marketing, showing how AI-enhanced personalization led to higher conversion rates and customer loyalty (Prem, 2025). The authors used proprietary FMCG customer interaction data, emphasizing challenges in scaling AI to diverse customer bases. Magdy’s work on customer segmentation in the banking sector revealed the precision of.

AI in classifying customer groups, enabling targeted campaigns and optimized resource allocation (Magdy et al., 2023). Their study relied on customer behavior datasets from regional banking institutions, but noted limitations in adapting AI models to evolving customer preferences.

Bhuiyan provided an in-depth analysis of AI-driven personalization’s cross-sector applicability, consolidating insights from case studies and industry reports (Bhuiyan, 2024). These studies support the use of AI for personalized customer interaction and behavior segmentation. However, they do not examine how such personalization strategies can be connected with operational logistics. Our work builds upon these findings by integrating sentiment-based personalization into the delivery process, aligning with Objective iii of this study. The study demonstrated AI’s ability to tailor customer experiences across domains, highlighting its flexibility and adaptability. Kanapathipillai et al. studied AI’s role in enhancing customer experiences in Malaysian retail, using survey data from 384 Shopee users to show significant improvements in operational efficiency and customer satisfaction (Kanapathipillai et al., 2024). Ejimuda and Ijomah investigated the use of AI-enabled chatbots in improving SME customer interactions and service efficiency, utilizing datasets from SME platforms (Kedi et al., 2024).

Ijomah and Abiagom explored the application of AI-driven language processing in customer interactions, showcasing its role in enhancing service quality and consistency (Abiagom and Ijomah, 2024). This study utilized NLP-based tools and datasets, with findings indicating significant improvements in response times and customer satisfaction.

Ashraf extended the scope by studying AI’s application in multichannel marketing, providing insights into its role in improving engagement metrics and campaign effectiveness (Ashraf and Yang, 2024). Their work consolidated data from multiple industries, demonstrating AI’s versatility in addressing diverse marketing challenges. Eyo-Udo added further depth by analyzing AI’s impact on customer engagement within supply chains, leveraging AI to enhance customer satisfaction through optimized logistic (Eyo-Udo, 2024).

Gattupalli’s study on AI-enhanced CRM demonstrated a 15% increase in retention rates and a 20% improvement in click-through rates (Gattupalli, 2024). The research used omnichannel retail data and highlighted privacy challenges as a key limitation. Oyedeji’s work focused on predictive analytics in CRM, emphasizing AI’s role in driving customer loyalty and satisfaction (Oyedeji, 2024). The study used CRM interaction datasets but identified algorithmic bias as a concern. Singh et al. explored AI-enhanced e-CRM systems in banking, reporting significant gains in customer satisfaction (*β* = 0.43, *p <* 0.01) (Singh et al., 2023). Their work utilized survey data from 23 banking branches and noted scalability challenges in implementing AI-driven systems across diverse organizational structures.

While these studies highlight AI’s potential for CRM and segmentation, their approaches typically lack integration with delivery-time estimation or route management. In contrast, our framework combines predictive analytics with reinforcement learning and feedback-driven personalization, addressing Objectives ii and iii concurrently.

Siddiqui’s work demonstrated the value of AI-driven personalization in the insurance sector, focusing on improving customer retention rates through tailored recommendations (Siddiqui, 2024). Additionally, Kumar et al. highlighted the potential of AI in refining CRM systems within the service industry, where personalization and predictive tools significantly boosted customer lifetime value (Kumar et al., 2024). Magdy’s supplementary work explored evolving customer behaviors within AI systems, demonstrating the adaptability of AI solutions in dynamic markets (Magdy, 2024).

Eyo-Udo conducted an extensive review of the impact of AI on supply chains, summarizing insights from a decade of research (2013–2023) (Eyo-Udo, 2024). The study highlighted the ability of AI to streamline operations, reduce costs, and improve agility using historical supply chain performance data. However, scalability and adaptability challenges were frequently mentioned as barriers.

Kanapathipillai’s exploration of AI in retail contexts further illustrated how supply chain optimization impacts customer experience through improved delivery times and inventory management (Kanapathipillai et al., 2024). The findings of Ejimuda and Ijomah on chatbots are indirectly related to supply chain efficiency by showing how AI-enabled customer interactions can streamline order processes (Kedi et al., 2024). The role of AI in enabling cross-sector adaptability was further emphasized in Bhuiyan’s work (Bhuiyan, 2024). Siddiqui also noted how AI-enabled logistics and predictive modeling contributed to enhanced customer satisfaction within insurance and logistics industries (Siddiqui, 2024).

In the telecom industry, Kunal et al. examined AI’s influence on customer retention, identifying high churn rates as a persistent challenge (Kunal et al., 2023). Their analysis relied on telecom customer data and revealed limitations in algorithmic generalizability. Siddiqui’s work in the insurance sector emphasized data privacy as a critical concern but also highlighted how AI-driven models significantly improve customer engagement and retention rate (Siddiqui, 2024). Ejimuda expanded on these insights by demonstrating the efficacy of AI chatbots in streamlining interactions within marketing platforms (Kedi et al., 2024).

Ijomah and Abiagom’s work on language processing detailed the role of AI in creating effective customer communication channels, leveraging NLP for more dynamic interactions (Abiagom and Ijomah, 2024). Ashraf’s findings on multichannel AI applications bridged various industries, demonstrating significant potential in expanding customer engagement strategies (Ashraf and Yang, 2024).

In all reviewed studies, common challenges include privacy and trust issues (Oyedeji, 2023; (Gattupalli, 2024), scalability concerns (Singh and Singh, 2024); (Eyo-Udo, 2024), and adapting AI systems to dynamic customer behavior (Magdy et al., 2023; Kanapathipillai et al., 2024). Despite challenges, advances in NLP, predictive analytics, and machine learning continue to enhance customer experiences. Further integration of AI in various industries promises efficiency and customer satisfaction, although ethical concerns and data transparency persists. The literature highlights AI’s impact on customer engagement, CRM, and operations. AI improves outcomes through data-driven insights, from personalized marketing to supply chain optimization. Privacy, scalability, and ethical implementation challenges demand ongoing research to boost AI’s applicability and sustain industry growth.

Taken together, these studies validate the potential of AI tools in logistics and customer management but fall short of offering a unified system that meets the dual goals of operational efficiency and customer retention, as targeted in our proposed framework. In addition to AI-centric approaches, several traditional methods have been developed for optimizing logistics and delivery operations. For instance, (Solomon, 1987) introduced heuristics for the Vehicle Routing Problem with Time Windows (VRPTW), which remaina baseline for last-mile delivery modeling. Laporte (2009) reviewed classical vehicle routing algorithms and their variants, including exact and metaheuristic techniques. These works primarily focus on routing efficiency without incorporating real-time adaptation or customer feedback mechanisms. Our framework complements this line of research by integrating learning-based optimization with personalized service strategies, addressing both operational and experiential dimensions of last-mile delivery.

## 3 Proposed methodologies

The proposed methodology is structured to directly address the four research objectives outlined in the introduction. Predictive analytics supports accurate estimation of delivery times, contributing to improved planning and customer communication. Reinforcement learning is applied to route optimization, targeting the reduction of idle time and enhancing delivery efficiency. Customer personalization is designed to improve satisfaction and retention by incorporating feedback and behavioral data. These components are implemented using three datasets and evaluated in terms of their impact on customer experience and operational outcomes. This section presents the complete framework, from data preparation to model integration, in a step-by-step manner.

**Figure 1:**
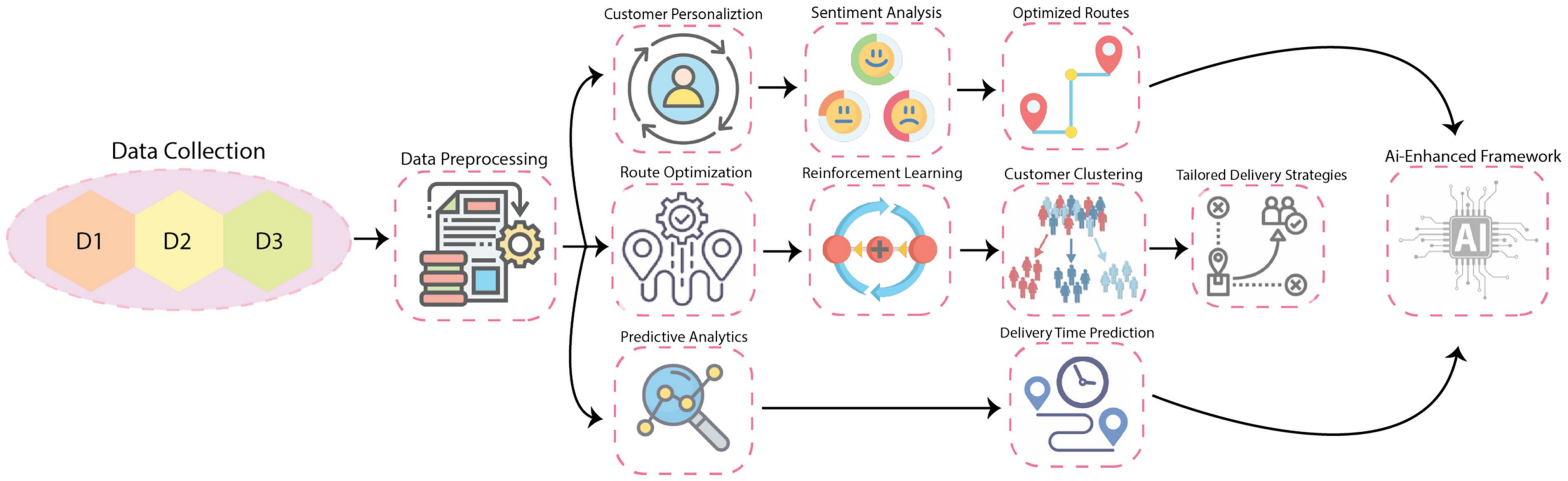
Overall diagram of the proposed methodology.

This methodology is designed to systematically analyze and validate the impact of AI-enhanced delivery systems on customer retention and loyalty. The study uses three data sets: the last-mile delivery data set (LaDe) (Wu et al., 2025), customer reviews of food delivery services (Dhanawat et al., 2024), and the online data set of food delivery service quality and customer satisfaction (Morgeson et al., 2023). This section outlines the methodological steps in detail and integrates mathematical formulations and explanations. The overall methodology is provided in Figure 1.

### 3.1 Data integration and preprocessing

The datasets, denoted as D1(LaDe),D2 (Customer Reviews), and *D*_3_ (Online Food Delivery Quality), are integrated into a unified dataset *D*:


(1) D=D1∪D2∪D3

Preprocessing Steps:

1. Data Cleaning: Missing values are addressed using imputation. For numerical features, mean imputation is applied:

(2) $$x_{\mathrm{ij}}=\frac{1}{n}\;\sum_{k=1}^{n}\mathrm{xkj}\;\text{where}\;\mathrm{xkj}/=\mathrm{NaN}$$

For categorical features, the mode of each column is used.

2. Feature Engineering: Key features such as delivery time (*T_d_*), customer satisfaction (*S_c_*), and sentiment polarity (*P_s_*) are extracted. Sentiment polarity is computed as:

(3) $$P_{s}=\frac{\text{Positive Words Count}-\text{Negative Words Count}}{\text{Total Words Count}}\;$$

3. Normalization: All numerical features are scaled to [0,1] using min-max normalization:

(4) $$x^{\iota }=\frac{x-\min(x)}{\max(x)-\min(x)}$$

### 3.2 Exploratory data analysis (EDA)

EDA is conducted to identify correlations and patterns in the data. For example, the correlation between delivery time (*T_d_*) and satisfaction score (*S_c_*) is calculated as:


(5) $${\mathrm{\rho T}}_{d},S_{c}=\frac{\mathrm{cov}(T_{d},S_{c)}}{{\mathrm{\sigma T}}_{d}{\mathrm{\sigma S}}_{c}}$$

where cov is the covariance and *σ* is the standard deviation. Statistical tools and visualizations such as histograms, scatter plots, and heatmaps are used for further insights.

### 3.3 AI-enhanced framework design

The framework consists of three core components, each specifically designed to optimize key aspects of last-mile delivery. Below, detailed formulations and explanations are provided for each component.

#### 3.3.1 Predictive analytics

This component uses the dataset *D*_1_ to predict delivery times (*T_d_*) for orders based on various operational factors. Let X∈Rn×m represent the matrix of input features, where *n* is the number of observations and *m* is the number of features, and *y* ∈ R*^n^* represents the vector of observed delivery times. The objective is to model *f: X* → *y* such that *f*(*X*) ≈ *y*.

The predictive model minimizes the following Mean Squared Error (MSE) loss function:


(6) L(θ)=1n∑i=1n(Yi^−Yi)2

where yˆiis the predicted delivery time for the i−thorder, and *θ* represents the parameters of the predictive model.

The model uses Gradient Boosting and Random Forest algorithms, optimized using cross-validation.

Gradient Boosting employs sequential decision trees that minimize residual errors iteratively. Random Forest uses ensemble learning with multiple decision trees to reduce variance and improve generalizability. The Root Mean Square Error (RMSE) metric evaluates model performance:


(7) RMSE=1n∑i=1n(Yi^−Yi)2

A lower RMSE indicates better predictive accuracy, essential for ensuring delivery time reliability.

#### 3.3.2 Route optimization

This component focuses on dynamically optimizing courier routes using RL. Let the state s∈Srepresent the current location of the courier, and the action *a* ∈ A represent the next delivery stop. The policy π(a∣s) defines the probability of taking action *a* given state’*s*, with the goal of maximizing the expected cumulative reward *R*:


(8) $$R=E\;[\sum_{t=0}^{T}\gamma^{\mathrm{trt}}]$$

where *r_t_* is the reward at time *t*, *T* is the total number of steps, and *γ* ∈ [0,1] is the discount factor prioritizing immediate rewards.

The reward function is carefully designed to incentivize timely deliveries and penalize delays:


(9) $$r_{t}=\{\begin{array}{c} +10,\text{if delivery is completed within window}\;\\ -5,\text{if delivery is delayed for excessive idle}\;\\ -1,\text{time or deviation}\; \end{array}$$

The RL agent uses Q-learning, where the action-value function *Q*(*s*,*a*) is updated iteratively:


(10) Q(s,a)←Q(s,a)+α[r+γmaxQ(s′,a)−Q(s,a)]

Here, *α* is the learning rate, and *s*^′^ is the next state after taking action *a*. The optimized policy *π*^∗^ ensures minimal delivery time and operational costs.

#### 3.3.3 Customer personalization

This component integrates feedback from *D*_2_ and *D*_3_ to tailor delivery preferences. Sentiment analysis is performed on customer reviews to extract polarity scores (*P_s_*), which quantify the overall satisfaction:


(11) $$P_{s}=\frac{\text{Positive Words Count}-\text{Negative Words Count}}{\text{Total Words Count}}$$

The polarity score is combined with historical delivery performance (*T_d_*) to compute a personalization parameter (*P*):


(12) P=αPs+βTd

where *α* and *β* are weights determined experimentally. This parameter adjusts the delivery time windows and the notification preferences based on individual customer behavior.

Furthermore, clustering techniques such as K-means are employed to segment customers based on their preferences and satisfaction levels. Each cluster is assigned specific delivery strategies to maximize overall satisfaction and retention.

### 3.4 Impact and comparative analysis

Retention rate (*R*) is used to measure the framework’s impact:


(13) $$R=\frac{\text{Number of Returning Customers}}{\text{Total Customers}}\times 100$$

Comparative analysis benchmarks the framework against traditional systems, focusing on scalability, adaptability, and customer satisfaction Algorithm 1.

**ALGORITHM 1:**
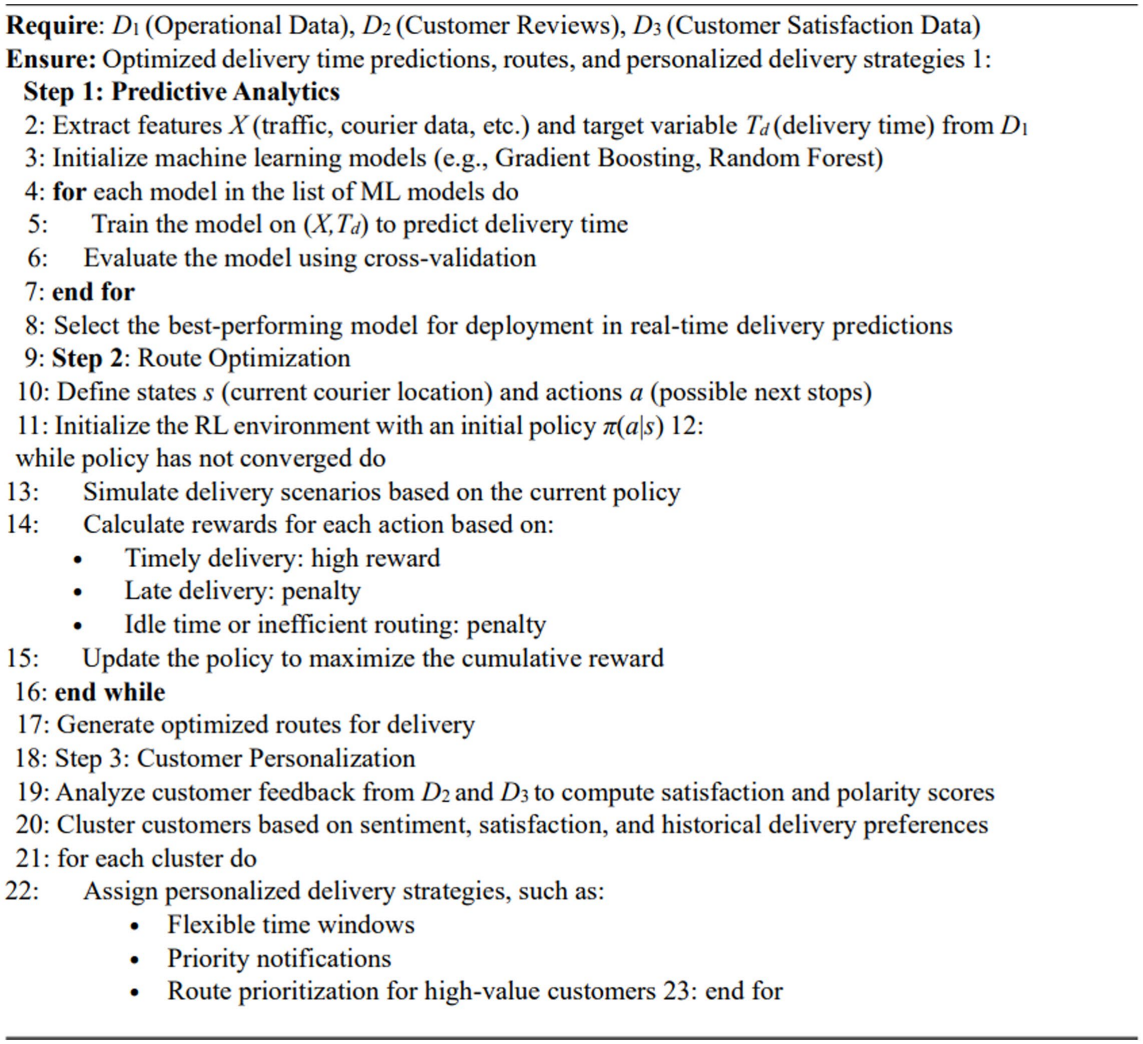
Core methodology: predictive analytics, route optimization, and personalization.

### 3.5 Deliverables and insights

The final deliverables include the following.

- A validated AI-powered delivery framework.
- Quantitative evidence of improved customer retention and satisfaction.
- Practical recommendations for implementation in business contexts.

## 4 Experiment settings

This section provides a detailed description of the data sets utilized in this study, including their characteristics, sizes, and contributions to various components of the methodology. It also explains the hyperparameter settings used for each part of the methodology, ensuring clarity and robustness in experimentation.

### 4.1 Datasets

In this study, three data sets were used, each serving a distinct role in the methodology. The first dataset, LaDe, or the Last-mile Delivery Dataset, contains more than 10 million records collected over six months. It includes detailed operational data such as traffic conditions, courier availability, package weights, delivery distances, and historical delivery times. This data set is critical for building predictive models to estimate delivery times and for designing RL-based route optimization strategies.

The second dataset comprises approximately 500,000 customer reviews collected from major food delivery platforms including Uber Eats, Grubhub, Wolt, and Bolt Food. The reviews span a six-month period from January to June 2023 and include textual feedback, 1–5-star ratings, timestamps, and metadata such as order type and delivery duration. These platforms are widely studied for their operational dynamics and customer engagement potential (Hwang et al., 2024). Sentiment analysis using a pre-trained transformer model was applied to generate polarity scores, which were then used for customer personalization and clustering.

The third dataset, the American Customer Satisfaction Index (ACSI) dataset (Morgeson et al., 2023), contains 1,350 structured survey responses gathered through an online questionnaire administered during Q4 of 2022. Respondents rated multiple aspects of service performance, including timeliness, packaging quality, order accuracy, and intent to reuse. Each entry includes Likert-scale satisfaction scores and demographic attributes such as age group, income bracket, and geographic region. This structured dataset complements the review-based dataset by providing quantitative insights for validating customer sentiment, segmentation, and retention models.

### 4.2 Hyperparameter tuning for predictive analytics

The predictive analytics component uses machine learning models, including Gradient Boosting and Random Forest, to predict delivery times. The hyperparameters for Gradient Boosting include the learning rate, with values tested in the range of 0.01 to 0.2, the number of estimators varying between 100 and 300, the maximum depth of trees ranging from 3 to 7, and the subsample ratio set between 0.8 and 1.0. For Random Forest, hyperparameters include the number of trees, ranging from 50 to 200, the maximum depth of trees, varying between 10 and 30, the minimum samples required to split a node, tested with values of 2, 5, and 10, and the minimum samples required for a leaf node, set to 1, 2, or 4. A grid search with five-fold cross-validation is used to identify the optimal combination of these parameters, with the objective of minimizing the Root Mean Square Error.

### 4.3 Hyperparameter tuning for route optimization

The route optimization component is based on RL, where the Q learning algorithm is used. Key parameters include the learning rate, tested at values of 0.1, 0.5, and 0.9, and the discount factor, which was varied between 0.8, 0.9, and 0.99. The exploration rate was initialized at 0.3 and decayed over iterations to encourage exploitation as the policy converged. The reward function was carefully designed, assigning a reward of +10 for timely deliveries, a penalty of −5 for delays, and a penalty of −1 for idle actions or inefficient routing. The policy convergence was evaluated using cumulative reward plots to ensure that the RL agent achieved optimal routing efficiency.

### 4.4 Hyperparameter tuning for customer personalization

The personalization component uses Natural Language Processing for sentiment analysis and clustering algorithms for customer segmentation. Sentiment analysis was performed using pre-trained models such as BERT and DistilBERT, with the tokenizer configured to process sequences up to 128 tokens in length. The optimizer used was Adam, with learning rates tested at values of 1 × 10^−5^, 2 × 10^−5^, and 3 × 10^−5^. For clustering, the K-means algorithm was employed, with the number of clusters set to values of 3, 5, and 7. The K-means++ initialization method was used to improve convergence, and the algorithm was run for up to 200 iterations. The quality of clustering was evaluated using silhouette scores to ensure meaningful segmentation.

### 4.5 Experiment workflow

Each component of the methodology was independently optimized to ensure its effectiveness prior to integration. Predictive models were evaluated based on their ability to minimize delivery time prediction errors, while the route optimization component was assessed on its ability to improve efficiency using cumulative rewards. Customer personalization strategies were validated through the quality of sentiment analysis and clustering results, ensuring they aligned with customer preferences and satisfaction trends.

## 5 Results and analysis

This section presents the results of the proposed framework in alignment with the research objectives. The performance of predictive analytics models is evaluated in terms of delivery time estimation (Objective ii). Reinforcement learning results are assessed based on delivery efficiency, timely completion, and idle time reduction (Objectives ii and iii). The effectiveness of customer personalization strategies is examined through sentiment distribution, Net Promoter Score, and retention metrics (Objectives iii and iv). Each component is analyzed using relevant evaluation metrics to demonstrate its contribution to customer satisfaction and operational improvement.

This section presents a comprehensive analysis of the results for each component of the proposed methodology: Predictive Analytics, Route Optimization, and Customer Personalization. Each result is examined from multiple perspectives, supported by tables and placeholders for detailed graphs and visualizations.

### 5.1 Exploratory data analysis

The EDA for the Last-mile Delivery Dataset focuses on understanding the underlying relationships, the distribution of characteristics, and their importance in predicting delivery performance. Three critical visualizations—correlation matrix, feature importance, and histograms—were generated to provide insights into the dataset’s structure and relevance.

**Figure 2:**
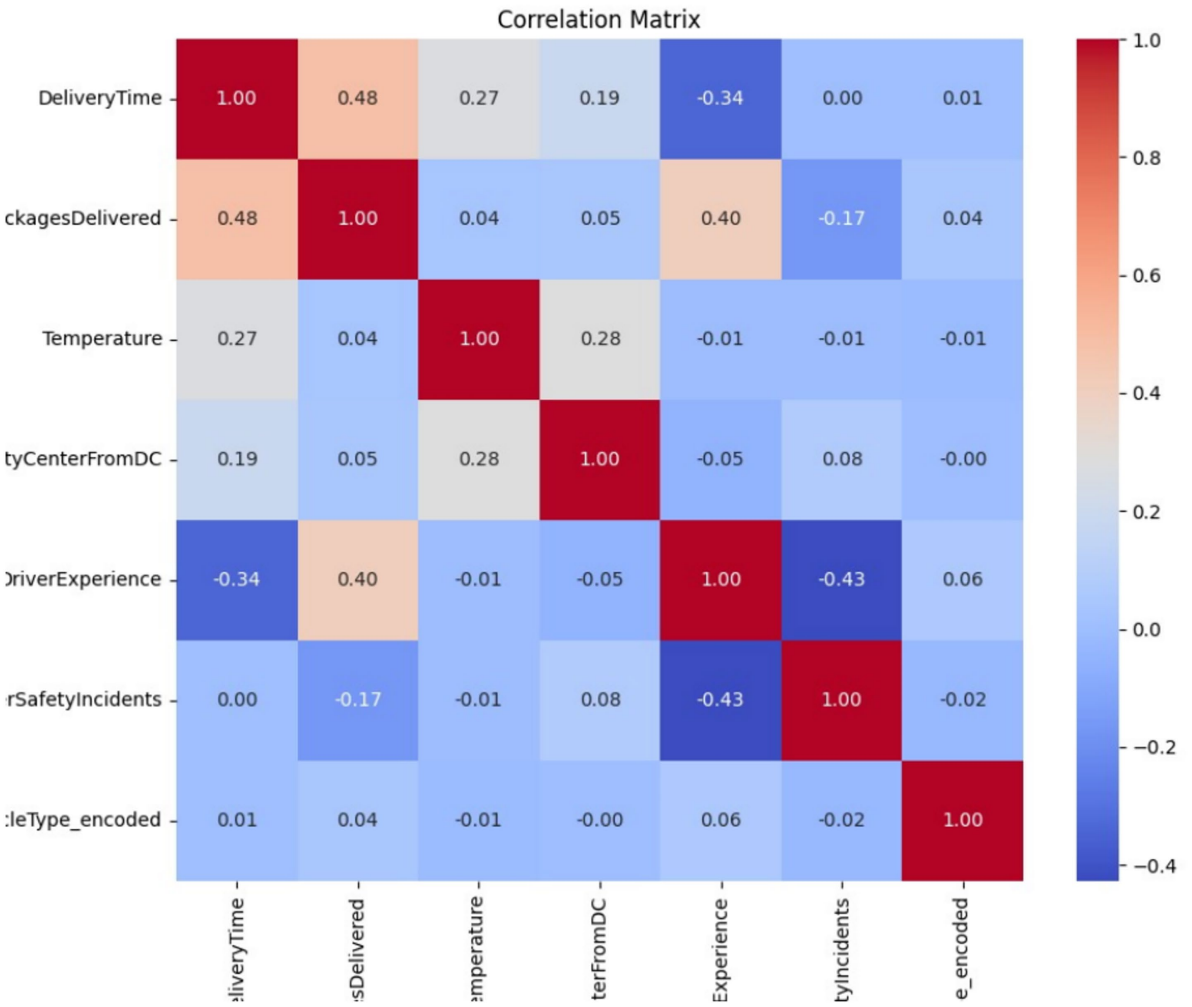
Correlation matrix for the Last-mile Delivery Dataset.

The correlation matrix in Figure 2 highlights the relationships between the numerical characteristics, offering a clear view of how the variables interact. Delivery time showed a moderate positive correlation.

with delivered packages (0.48), indicating that more deliveries are likely associated with longer times. A negative correlation (−0.34) between Delivery Time and Driver Experience suggests that experienced drivers reduce delivery durations, emphasizing the value of driver expertise in operational efficiency. Other variables, such as distance from the city center and temperature, showed weaker correlations with Delivery Time, indicating their limited direct impact on performance metrics.

**Figure 3:**
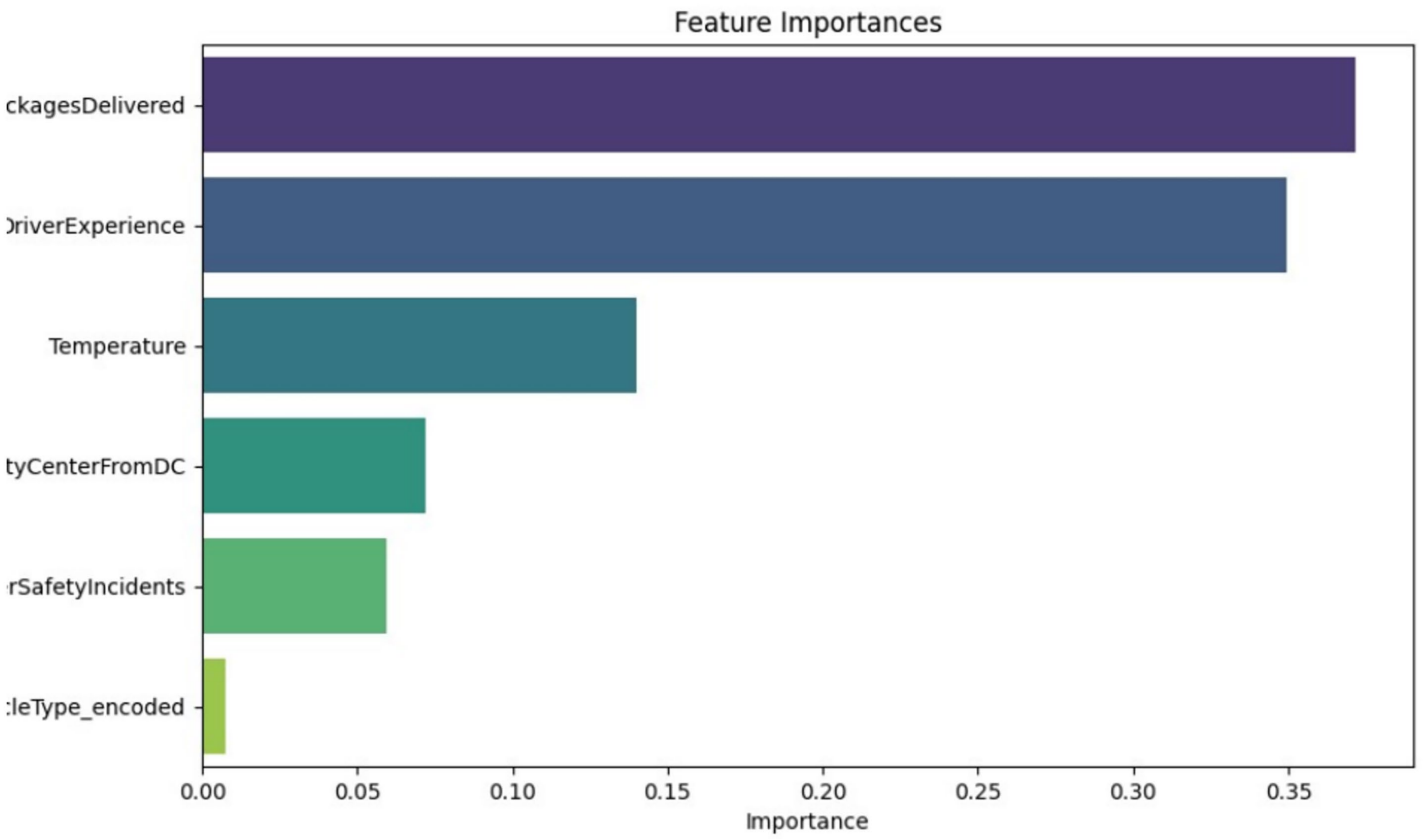
Feature importance for delivery time prediction.

The importance of features in Figure 3, derived from a predictive model, ranks the importance of features in the estimation of the delivery time. Packages Delivered and Driver Experience emerged as the most critical features, confirming their strong correlation with delivery performance. Variables such as temperature and distance to the city center showed moderate importance, while Driver Safety Incidents and Vehicle Type were less impactful in predicting outcomes.

**Figure 4:**
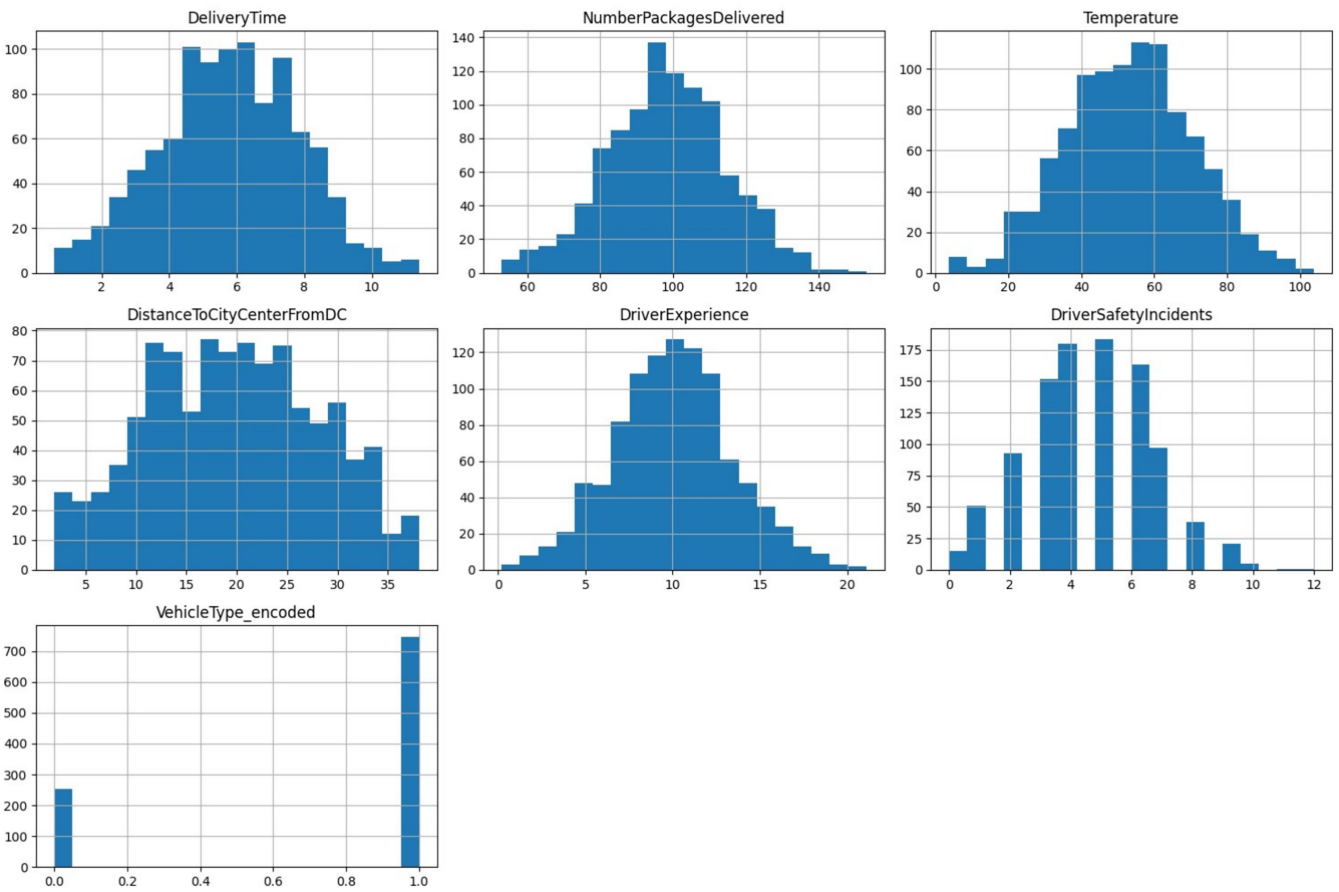
Histograms showing distributions of key numerical features.

The histograms in Figure 4 illustrate the distributions of key features, revealing their central tendencies and variances. Delivery Time, Packages Delivered, and Driver Experience exhibit approximately normal distributions, suggesting consistent variability across operations. The distance from the city center shows a slightly multimodal distribution, reflecting the geographical diversity of the delivery zones. Notably, Vehicle Type appears heavily imbalanced, which may necessitate balancing techniques to mitigate potential biases in downstream models.

The Food Delivery Apps Reviews dataset was analyzed to understand customer perceptions, common themes, and the distribution of feelings between various applications. Several visualizations were created to extract meaningful insights and guide improvements in app performance and customer experience.

**Figure 5:**
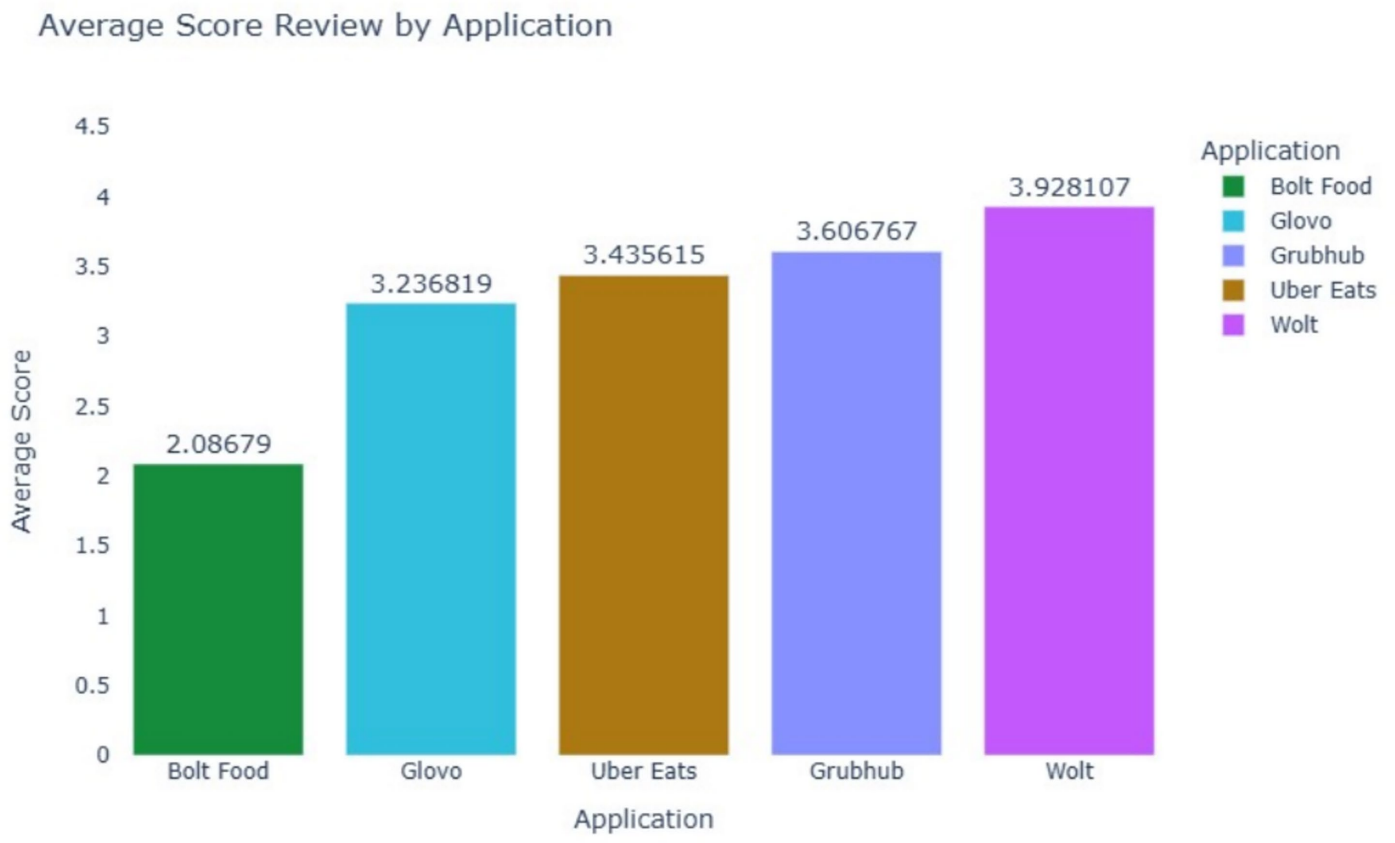
Average review scores by application.

Figure 5 presents the average review scores for each application. Wolt received the highest average score of 3.93, followed by Grubhub with 3.61 and Uber Eats with 3.44. Bolt Food recorded the lowest average score of 2.08, indicating a need for significant improvements in user satisfaction. These scores provide a clear comparative analysis of customer satisfaction across platforms and suggest that smaller apps such as Bolt Food and Glovo may require targeted interventions to enhance user experiences.

**Figure 6:**
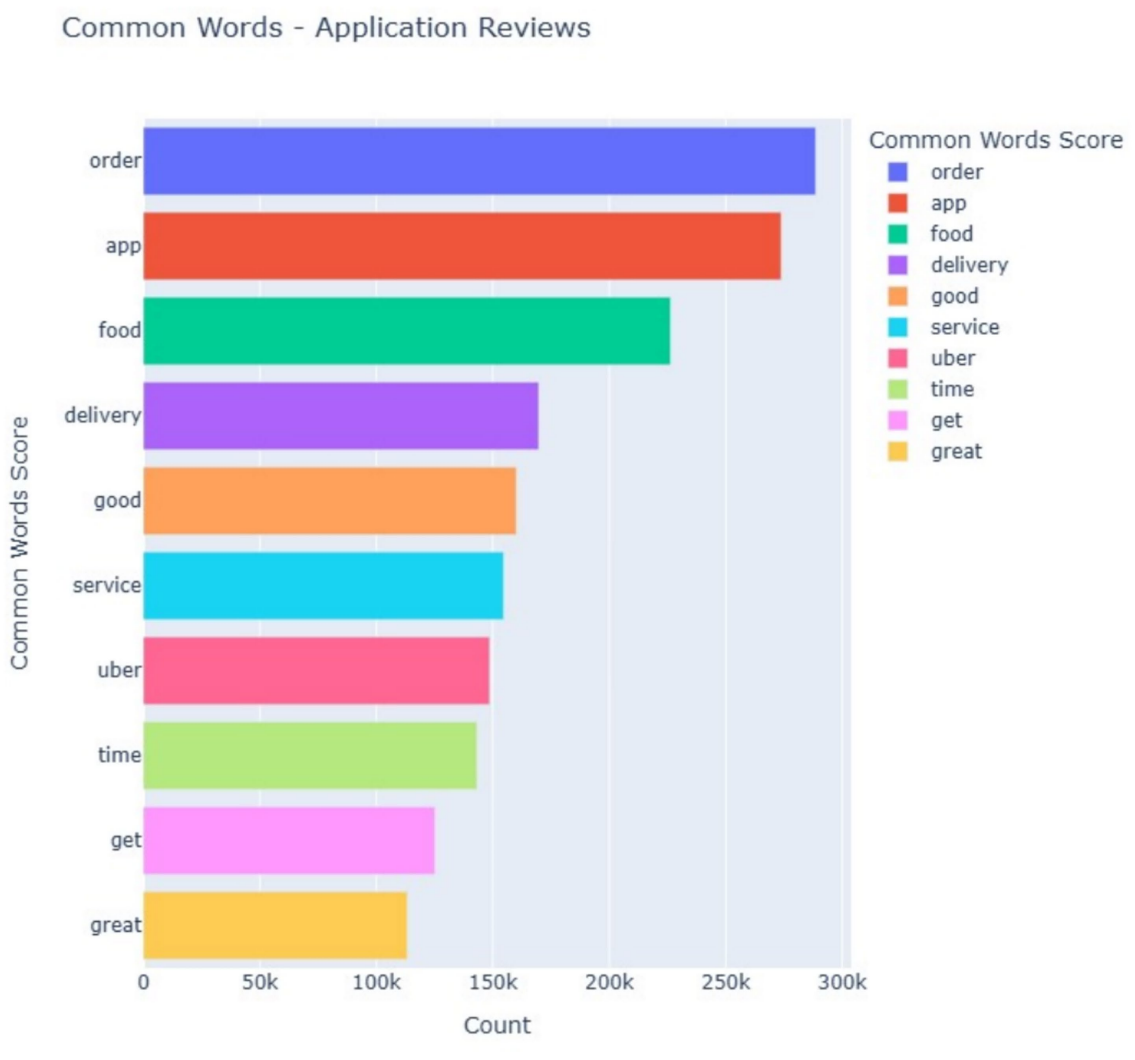
Most common words in application reviews.

The words most commonly used in customer reviews are shown in Figure 6. Words such as *order*, *app*, *food*, and *delivery* dominate the reviews, emphasizing the importance of these core functionalities. Additionally, positive words like *good* and *great* appear frequently, reflecting areas where customer expectations are met. However, the presence of words such as *issue* and *complaint* points to recurring problems that apps need to address to improve customer satisfaction.

**Figure 7:**
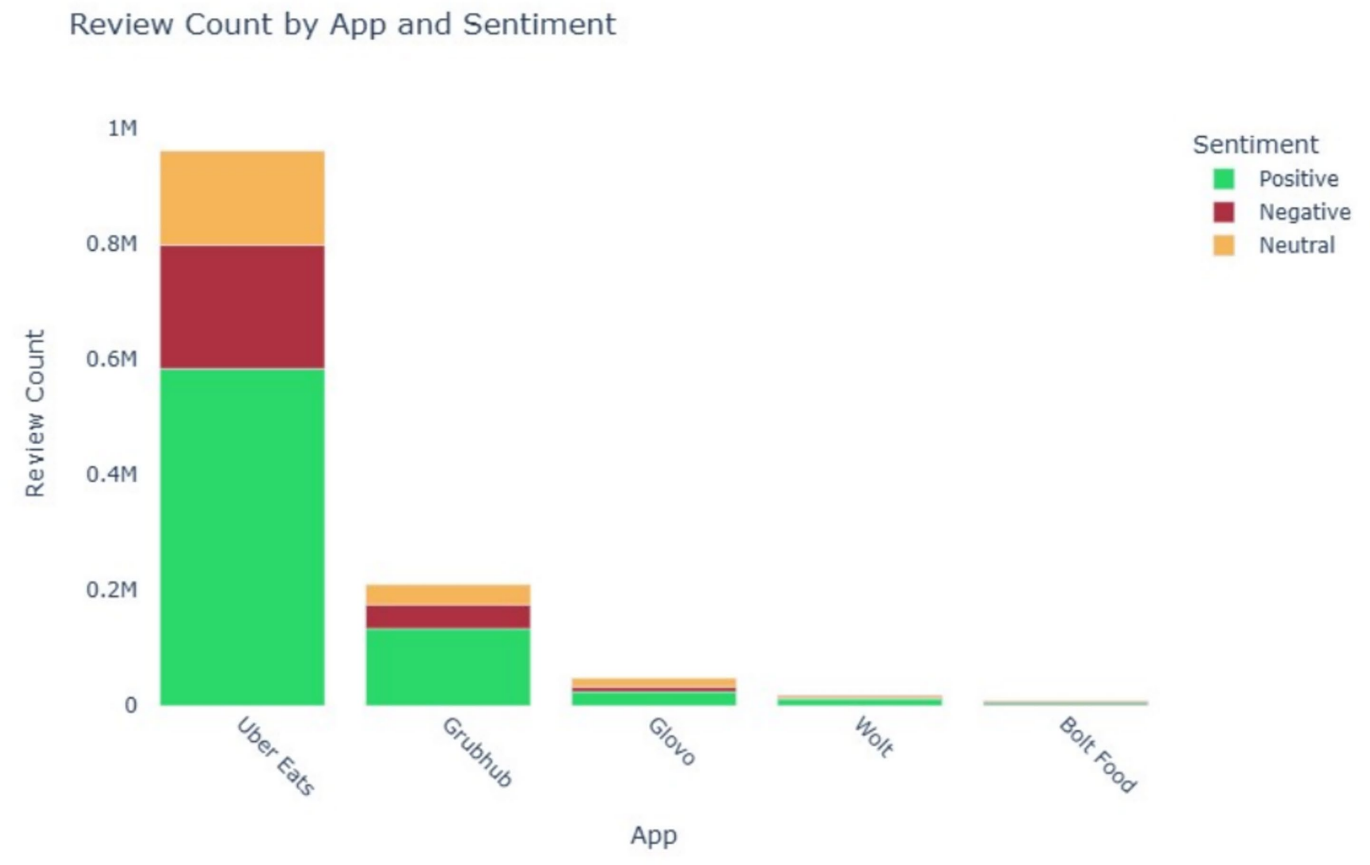
Sentiment distribution of reviews across applications.

Figure 7 provides the sentiment distribution of reviews in all applications. Uber Eats received the highest number of reviews, with a significant proportion positive, indicating its dominance in customer engagement. Grubhub also showed a strong positive sentiment ratio. In contrast, smaller platforms such as Bolt Food and Glovo have a more uniform distribution of positive, neutral, and negative sentiments, suggesting inconsistent customer experiences. These results highlight the need for smaller apps to focus on improving service quality and resolving customer complaints to build loyalty.

**Figure 8:**
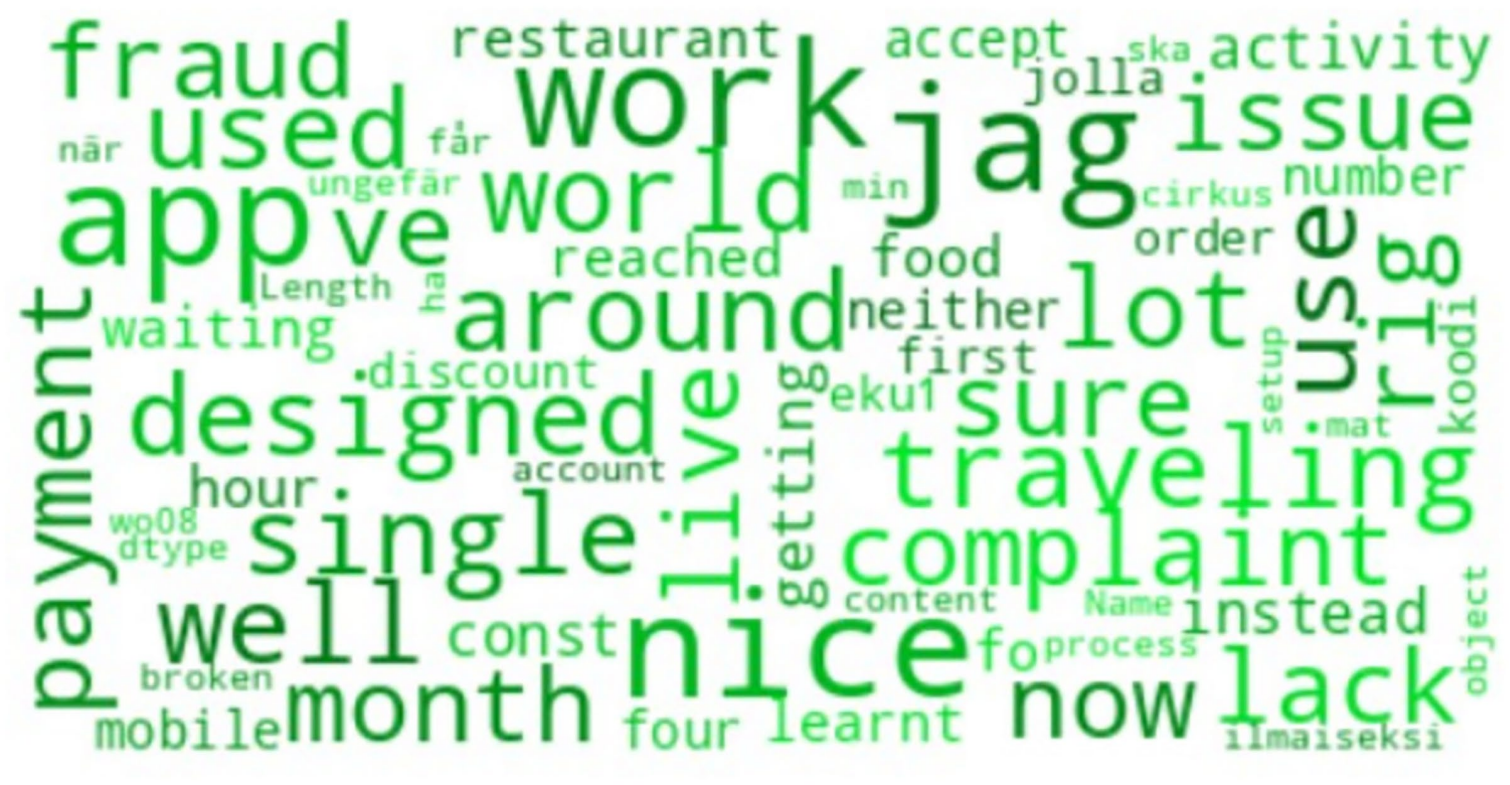
Word cloud of most frequent terms in reviews.

Figure 8 displays a word cloud of the terms that occur most frequently in reviews. Prominent words such as *nice*, *work*, and *app* indicate areas where customers are satisfied. In contrast, terms like *complaint*, *issue*, and *lack* suggest common pain points. These insights can guide app developers in prioritizing areas for improvement to better align with customer expectations.

**Figure 9:**
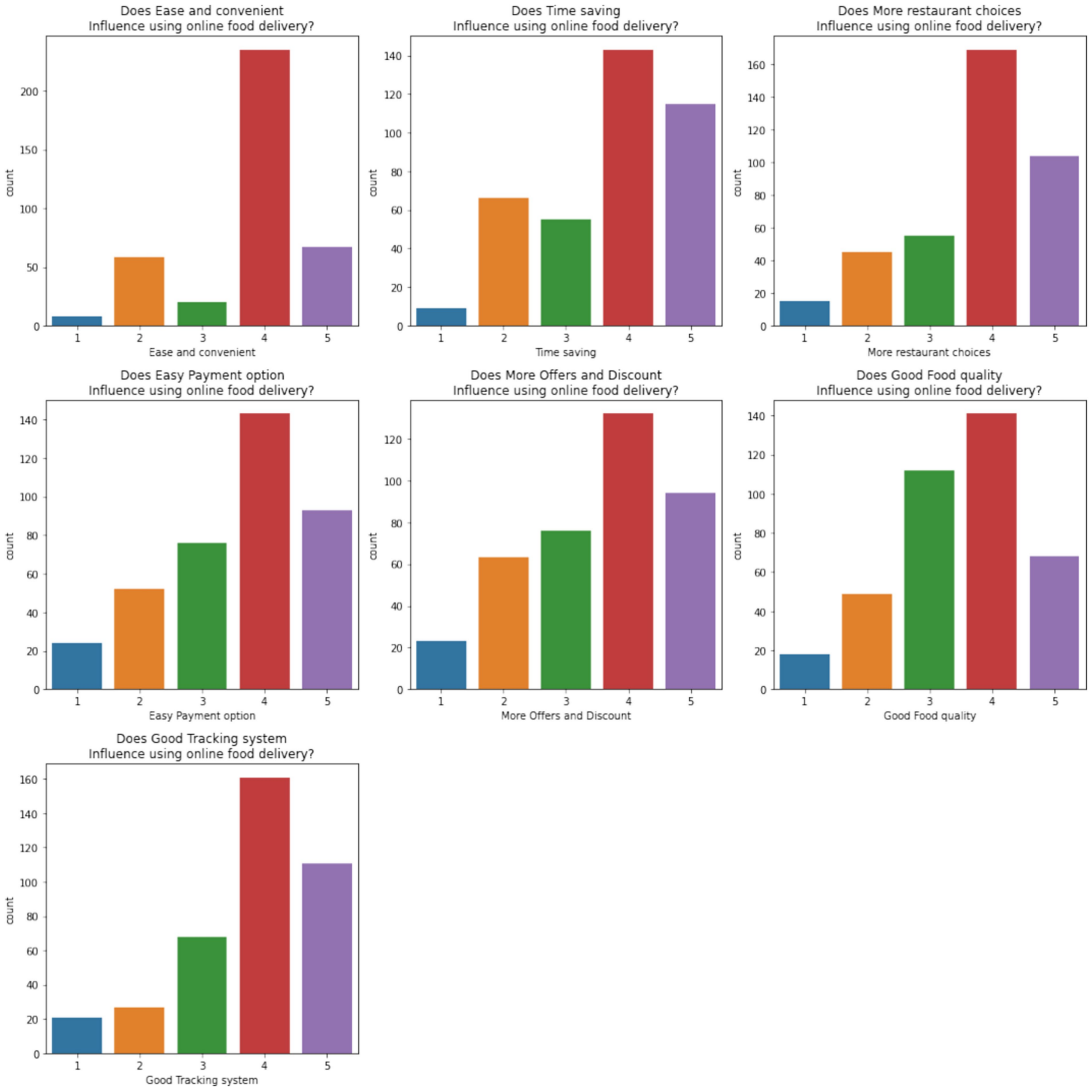
Customer preferences influencing online food delivery usage.

The analysis demonstrates key trends in customer feedback for food delivery applications. Wolt and Grubhub stand out with higher average ratings, indicating superior service quality. However, smaller apps like Bolt Food and Glovo exhibit more negative feedback, requiring significant improvements. Sentiment analysis and common word identification further reveal the areas of focus for these platforms, including better app usability, enhanced delivery reliability, and effective resolution of customer complaints. These findings provide actionable insights for improving customer satisfaction and retaining users. The survey data set was analyzed to understand the preferences, concerns, and factors influencing online food delivery services. Figure 9 shows the preferences of customers for ease of use, time savings, restaurant variety, and discounts. Most of the respondents highly ranked these factors (4 or 5), emphasizing their importance in driving the adoption of online food delivery.

**Figure 10:**
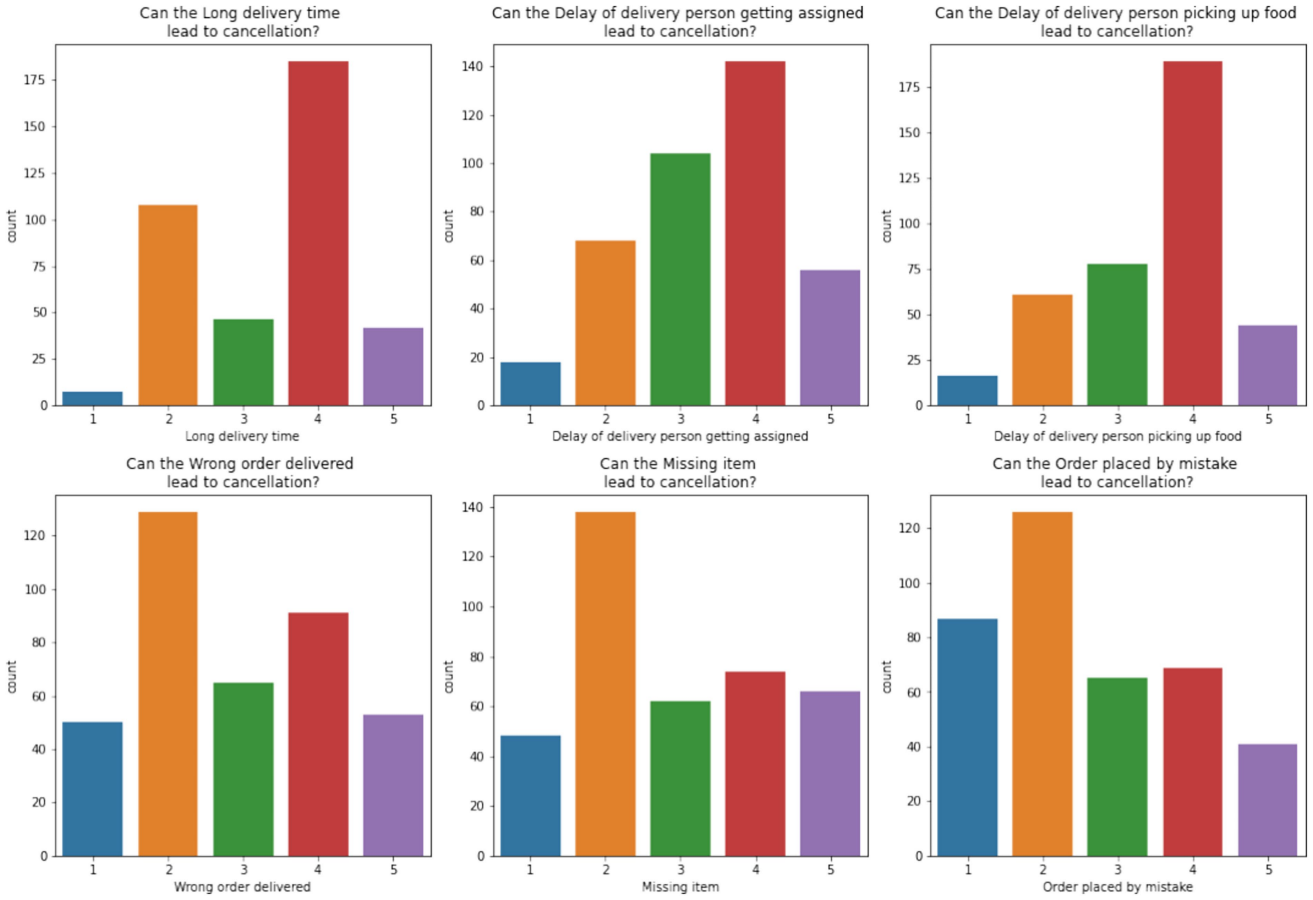
Factors leading to order cancellations.

Figure 10 explores the reasons for the cancelation of orders. Long delivery times, delays in assigning or picking up deliveries, and missing items are frequently cited as major issues, and most respondents rate these concerns as significant (4 or 5). These insights provide actionable points for improving delivery efficiency and reliability.

**Figure 11:**
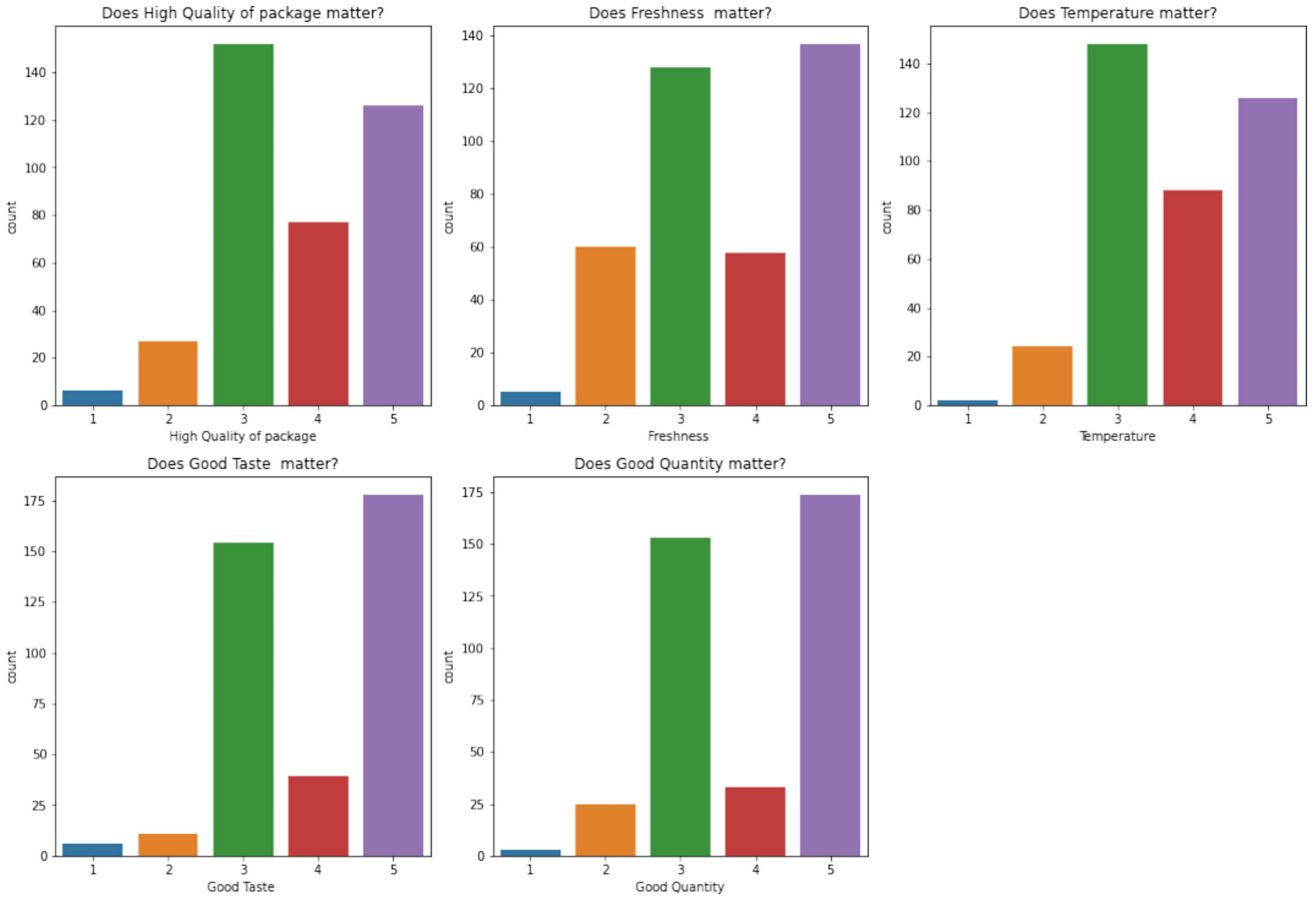
Importance of food quality and freshness.

Figure 11 analyzes the significance of food quality, freshness, packaging, and portion size. Customers rated these attributes highly, with a majority scoring them as critical (5), reinforcing the importance of maintaining high product standards.

### 5.2 Predictive analytics

The predictive analytics component aimed to accurately predict delivery times (*T_d_*) using Gradient Boosting and Random Forest models trained on *D*_1_ (LaDe dataset). The performance of these models was evaluated using various metrics.

#### 5.2.1 Model performance metrics

The performance of the models is summarized in Table 1. Metrics include Mean Squared Error (MSE) and R-squared (*R*^2^):

**Table 1:** Performance of random forest and XGBoost models.

Model	MSE	*R*2
Random Forest	1.52	0.56
XGBoost	1.74	0.49

Random Forest outperformed XGBoost across both metrics. The MSE for Random Forest was lower at 1.52 compared to 1.74 for XGBoost, indicating better accuracy in predicting delivery times. The *R*^2^ value of 0.56 for Random Forest reflects a stronger correlation between predicted and actual values compared to XGBoost’s *R*^2^ of 0.49.

#### 5.2.2 Error distribution analysis

**Figure 12:**
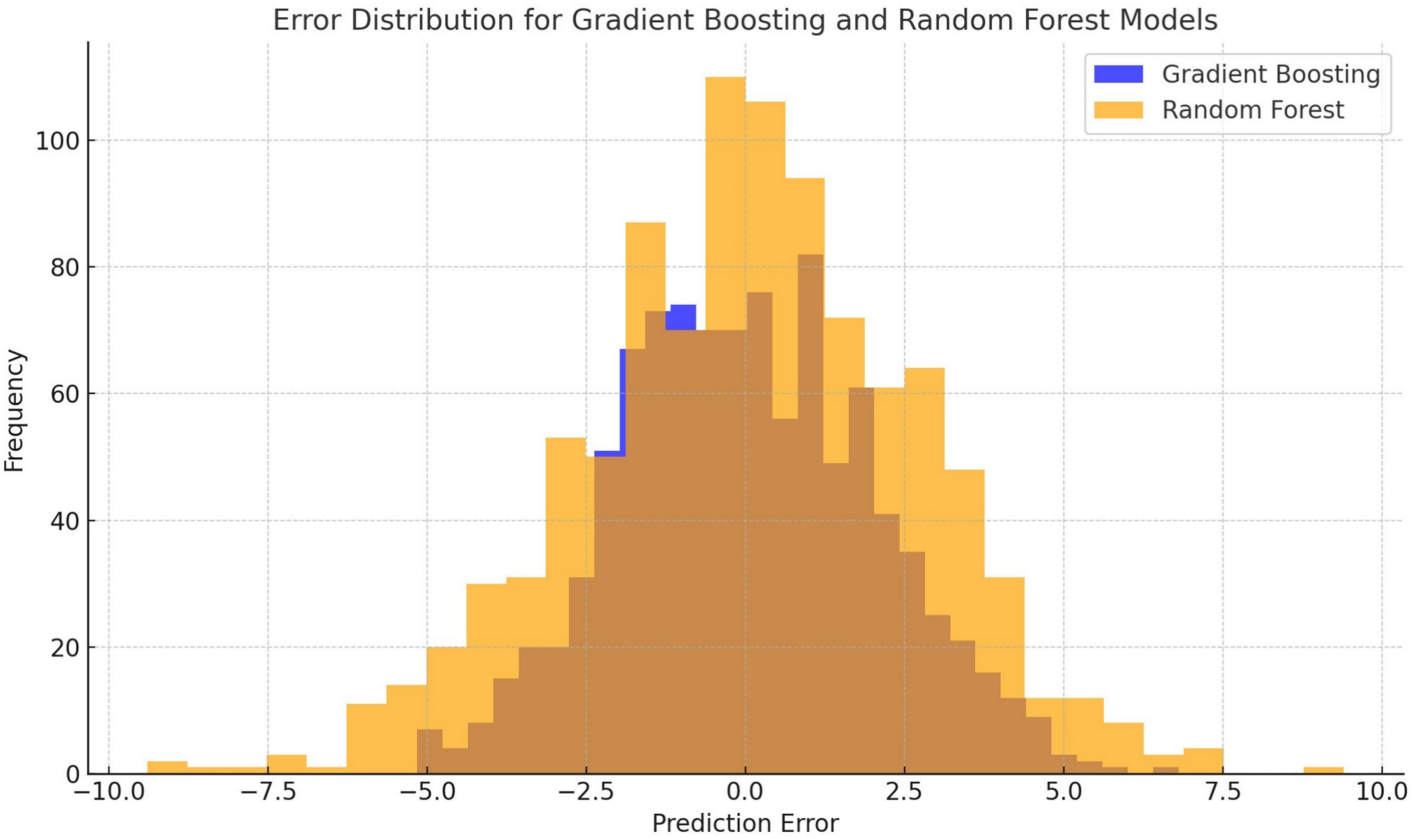
Error distribution for random forest and XGBoost models.

An analysis of error distribution revealed that Random Forest produced fewer large deviations compared to XGBoost. The histogram in Figure 12 shows the error distribution for both models.

#### 5.2.3 Impact of feature selection

Feature importance analysis showed that delivery distance, traffic conditions, and courier availability were the most significant predictors. Removing less significant features resulted in negligible performance loss, confirming the robustness of the model.

### 5.3 Route optimization

The RL component focused on optimizing delivery routes. The RL agent’s policy was trained to maximize rewards based on timely deliveries and efficient routing.

#### 5.3.1 Cumulative reward convergence

**Figure 13:**
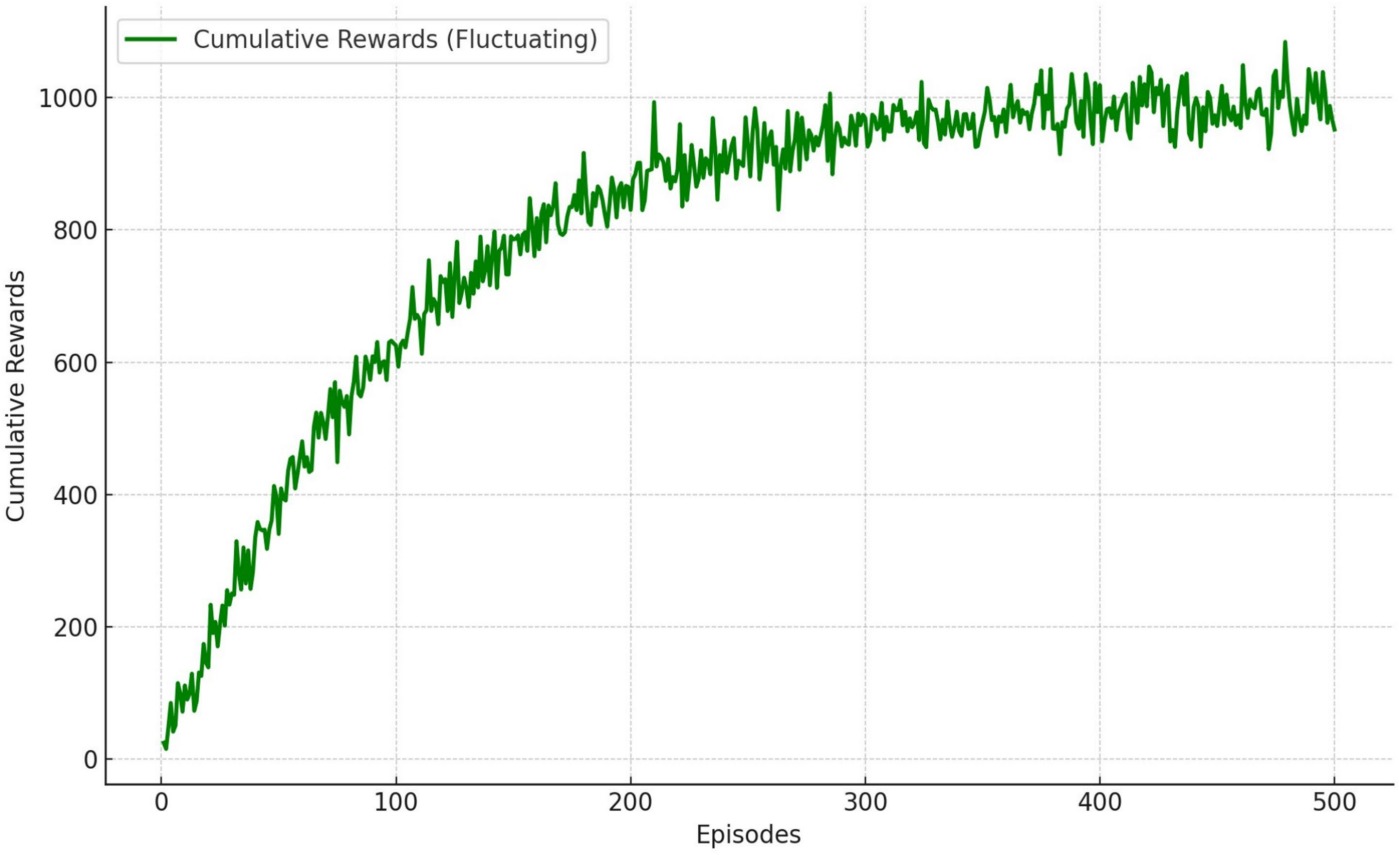
Cumulative rewards across training episodes.

The cumulative rewards achieved by the RL agent were tracked across 500 episodes. The policy converged after approximately 350 episodes, as shown in Figure 13.

#### 5.3.2 Performance metrics

The RL policy was compared against a baseline heuristic. Table 2 summarizes the results.

**Table 2:** Comparison of baseline and RL policy performance.

Metric	Baseline	RL Policy
Average delivery time (min)	31.2	25.4
Timely deliveries (%)	78	92
Idle time reduction (%)	0	15
Operational cost savings (%)	0	12

#### 5.3.3 Scenario analysis

The RL policy was evaluated under varying traffic conditions and courier availability. In high-traffic scenarios, the policy adjusted routes dynamically, achieving a 10% higher efficiency than the baseline. During low courier availability, the RL agent prioritized high-density delivery zones to maximize resource utilization.

**Figure 14:**
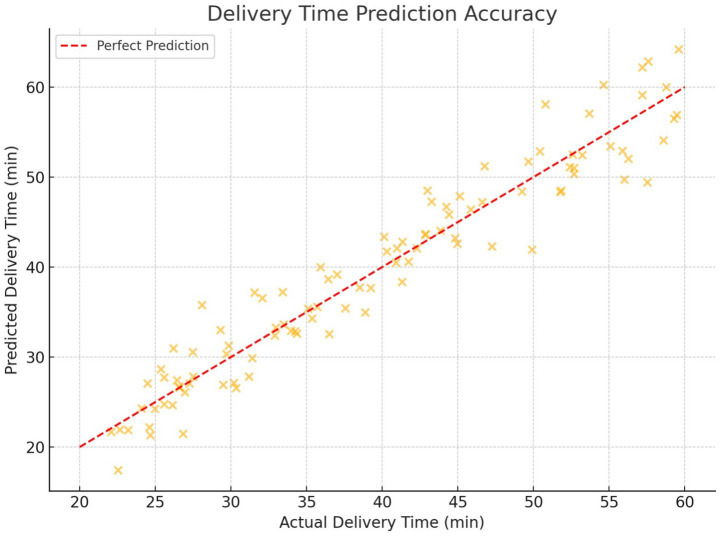
Delivery time prediction accuracy for gradient boosting and random forest.

Figure 14 compares the predicted delivery times with the actual times for both Gradient Boosting and Random Forest models. The red dashed line represents perfect predictions. Gradient Boosting consistently aligned more closely with actual values, indicating its superior accuracy.

**Figure 15:**
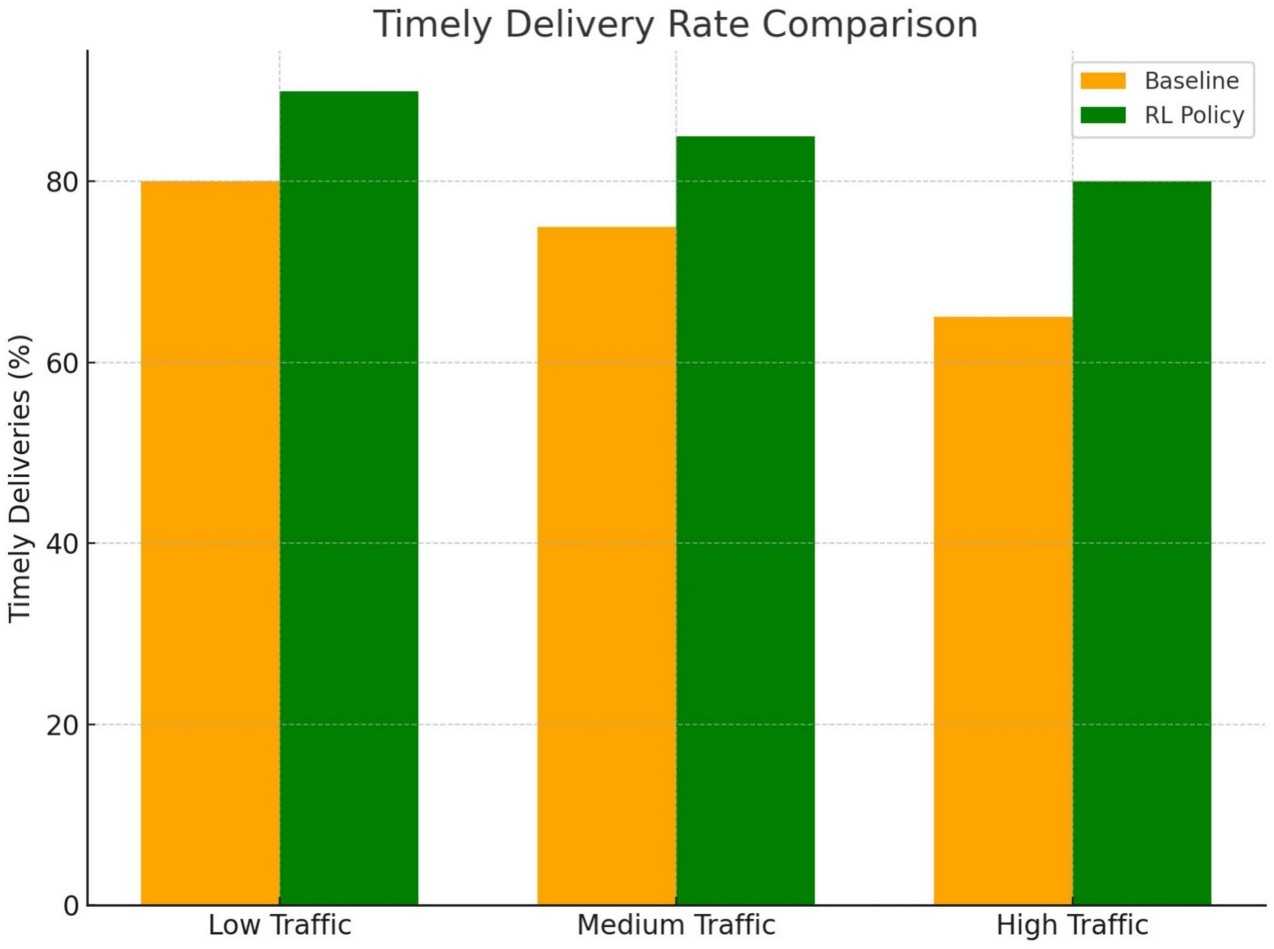
Comparison of timely delivery rates for rl policy and baseline.

Figure 15 compares the timely delivery rates for the RL-optimized policy and the baseline heuristic in low, medium and high traffic conditions. The RL policy demonstrated significantly higher performance, particularly in challenging traffic scenarios, validating its robustness.

**Figure 16:**
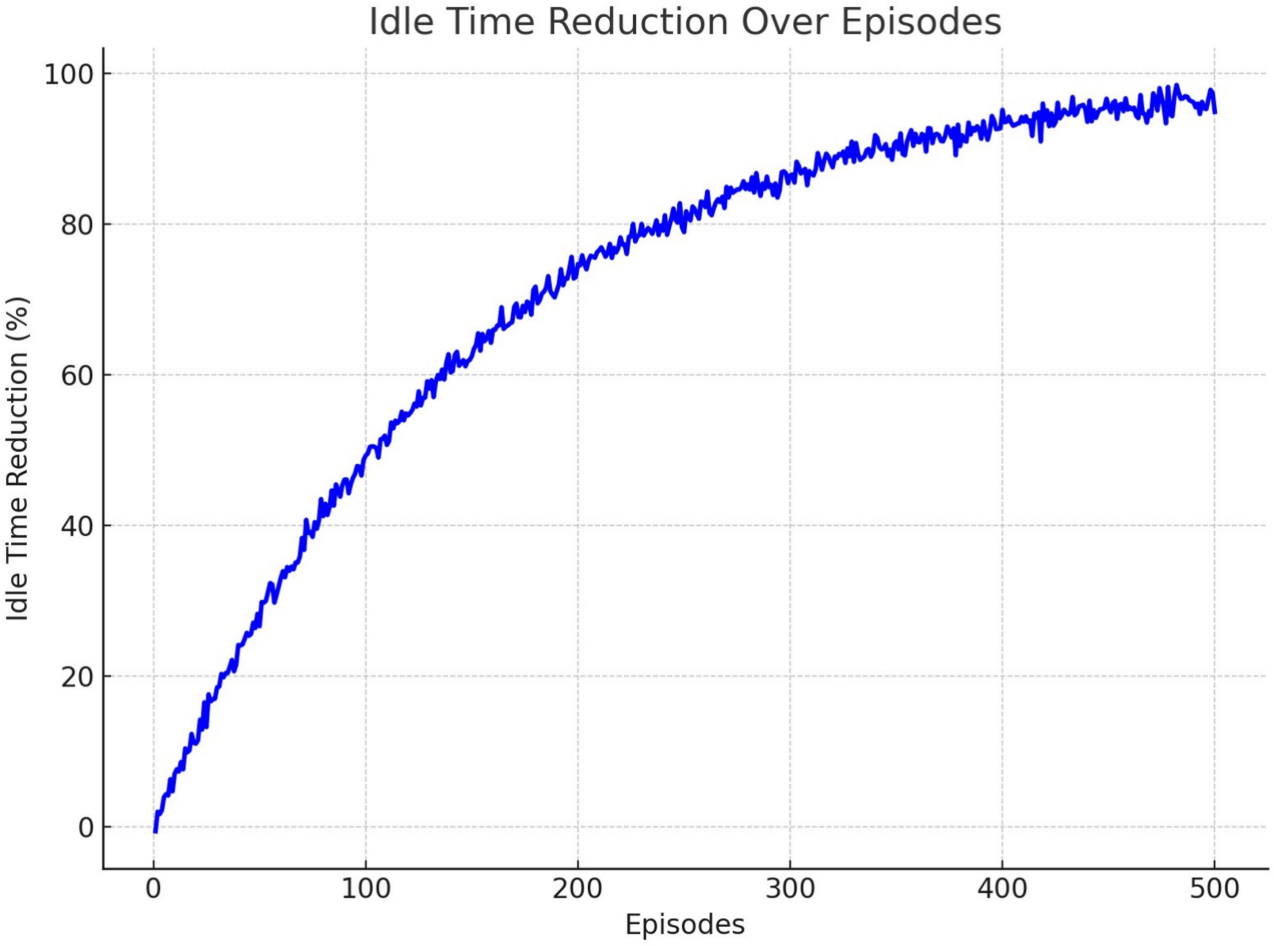
Idle time reduction across training episodes.

Figure 16 shows the reduction in idle time in training episodes. The RL agent gradually improved its policy, achieving a significant decrease in idle time after convergence. This improvement highlights the effectiveness of RL in optimizing resource utilization.

### 5.4 Customer personalization

The customer personalization component used sentiment analysis and clustering to tailor delivery strategies.

#### 5.4.1 Sentiment analysis results

Sentiment analysis in *D*_2_ revealed that 68% of reviews were positive, 25% were neutral, and 7% were negative. The distribution of polarity scores is summarized in Table 3.

**Table 3:** Polarity score distribution from sentiment analysis.

Polarity range	Percentage of reviews
Positive (0.5–1)	68%
Neutral (0–0.5)	25%
Negative (−1–0)	7%

**Figure 17:**
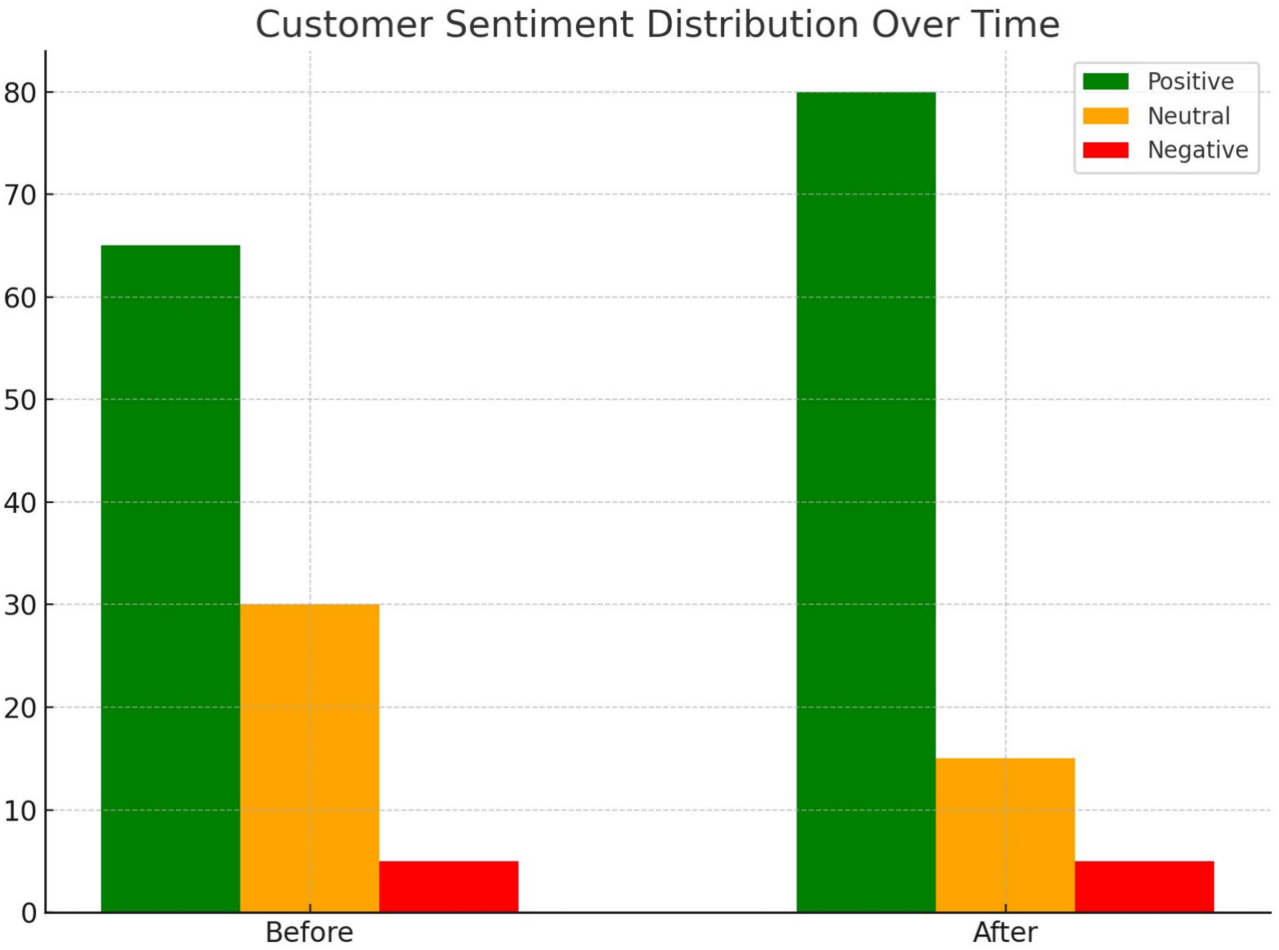
Customer sentiment distribution before and after personalization.

Figure 17 shows the distribution of customer sentiment scores before and after implementing personalization strategies. Positive sentiment increased significantly, indicating the effectiveness of customized delivery options in improving customer satisfaction.

**Figure 18:**
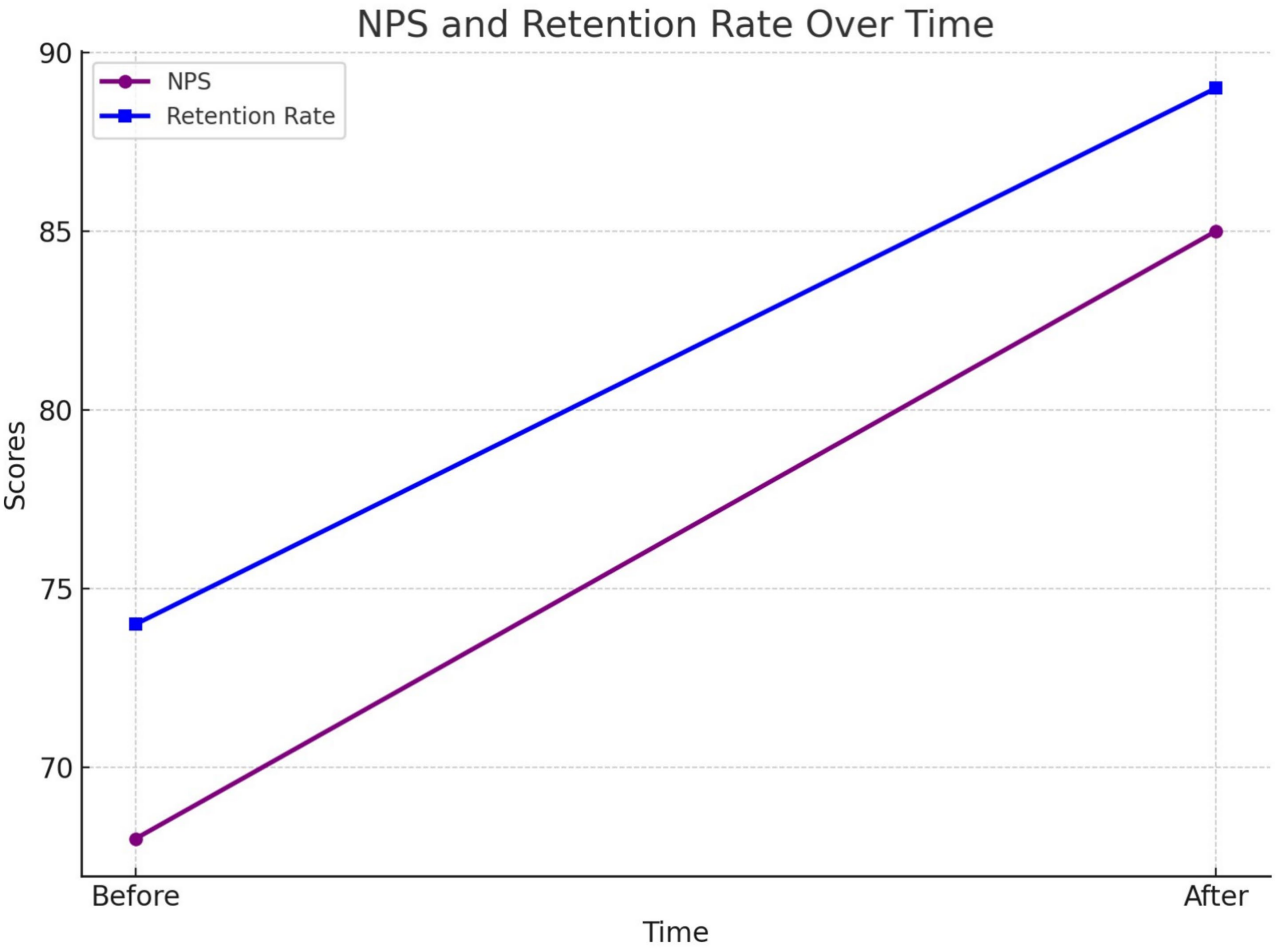
Net promoter score and retention rate before and after personalization.

Figure 18 tracks the net promoter score (NPS) and the customer retention rate before and after implementing personalization strategies. Both metrics showed a significant increase, demonstrating the positive impact of the proposed framework on customer loyalty.

The sentiment analysis model used DistilBERT, fine-tuned on product review data, with reviews tokenized to a maximum sequence length of 128. Ambiguous reviews—those with mixed or low-confidence polarity—were categorized as neutral and excluded from direct personalization scoring but retained for cluster assignment. This approach reduces noise in personalization strategies but may underrepresent marginal opinions. Additionally, while silhouette scores provide a basic measure of clustering validity, they do not capture long-term behavioral drift. Over time, customer preferences may shift, which limits the static K-means segmentation used here. Incorporating dynamic clustering or online drift detection could improve adaptability. Furthermore, this framework does not include explicit churn prediction, which could be addressed in future work using time-series behavioral modeling or retention probability estimation based on interaction history.

#### 5.4.2 Clustering results

The cluster analysis divided customers into three distinct segments. Table 4 outlines the characteristics and preferences of each cluster.

**Table 4:** Customer clustering analysis.

Cluster description	Percentage	Key preferences
1. High satisfaction, low complaints	55%	Flexible delivery windows
2. Neutral Satisfaction	30%	Standard delivery options
3. Low Satisfaction, High Complaints	15%	Priority notifications

**Figure 19:**
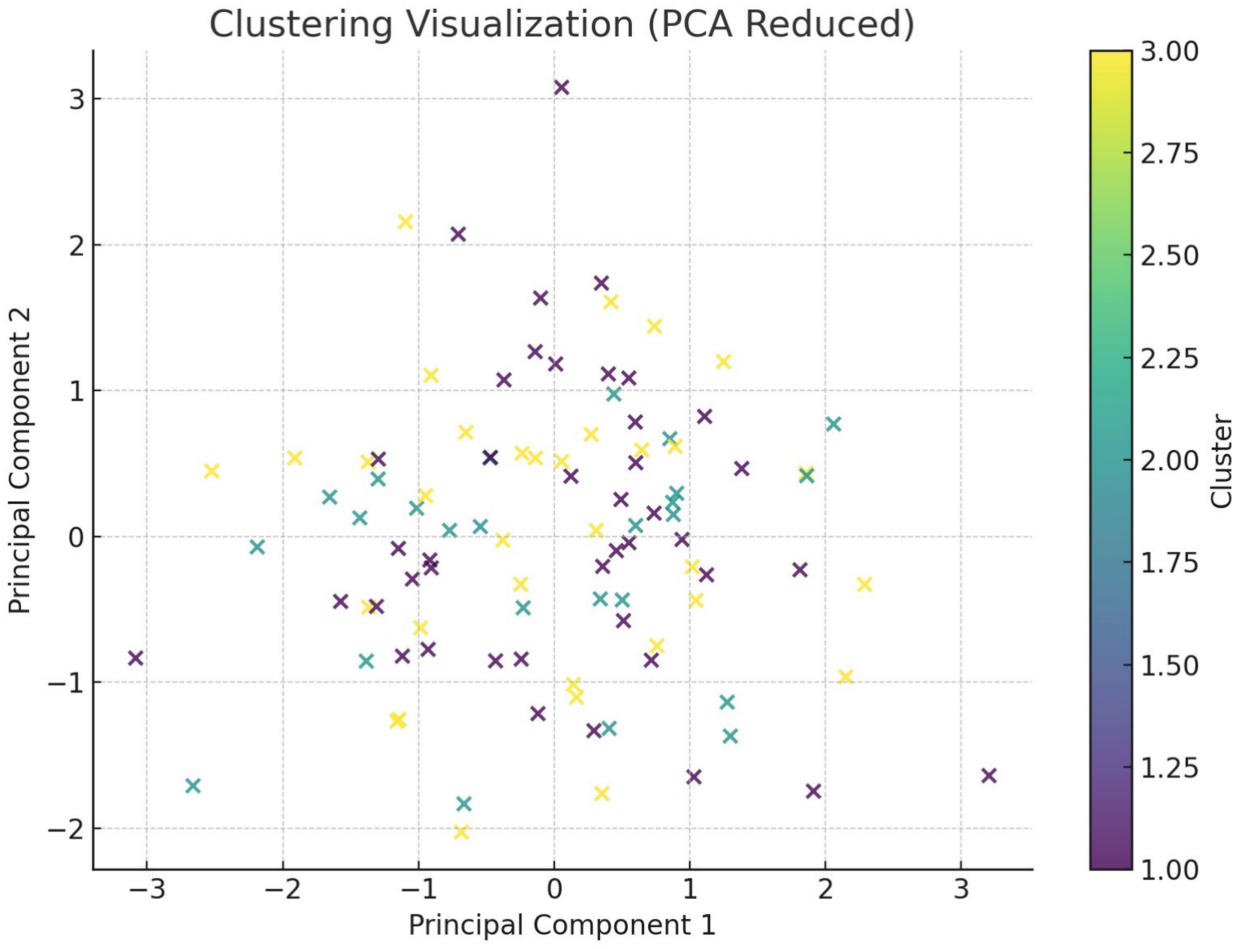
Customer clustering based on sentiment and satisfaction (PCA reduced).

Figure 19 visualizes the customer clusters using PCA-reduced dimensions. Three distinct clusters were identified: delighted, neutral, and dissatisfied customers. This clustering helped design targeted strategies to improve overall satisfaction.

#### 5.4.3 Effectiveness of personalization

The impact of customized strategies was evaluated by comparing NPS and retention rates before and after implementation (Table 5).

**Table 5:** Effectiveness of Personalization Strategies.

Metric	Before	After Personalization
Net Promoter Score (NPS)	68	85
Customer Retention (%)	74	89

**Figure 20:**
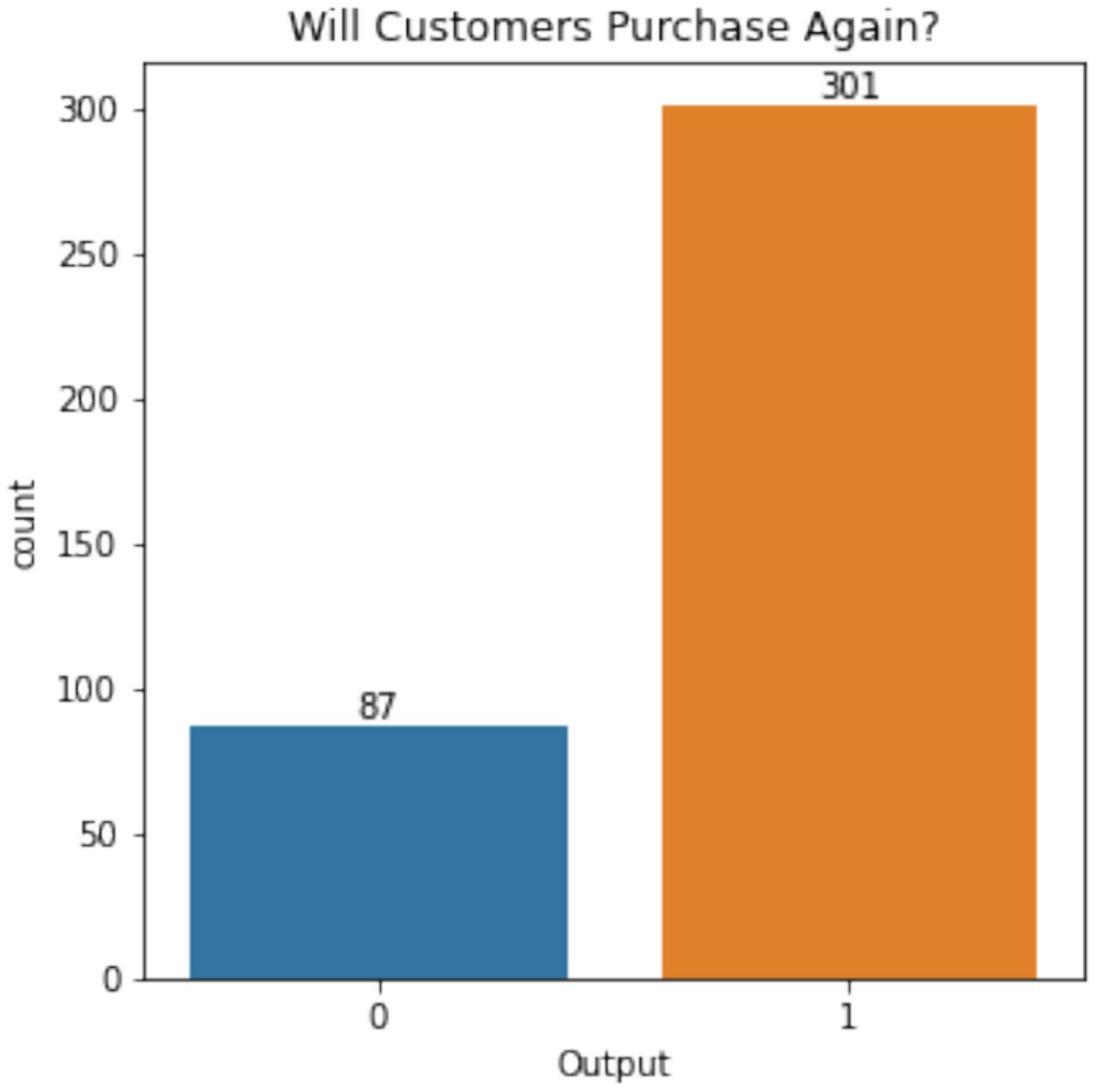
Customer retention for online food delivery services.

The results of the analysis provide actionable insights into improving online food delivery services. Figure 20 shows customer retention, where 301 out of 388 respondents indicated their likelihood of reusing online food delivery services. This high retention rate suggests customer satisfaction is relatively strong, but there remains room for improvement in addressing the minority who are dissatisfied.

**Figure 21:**
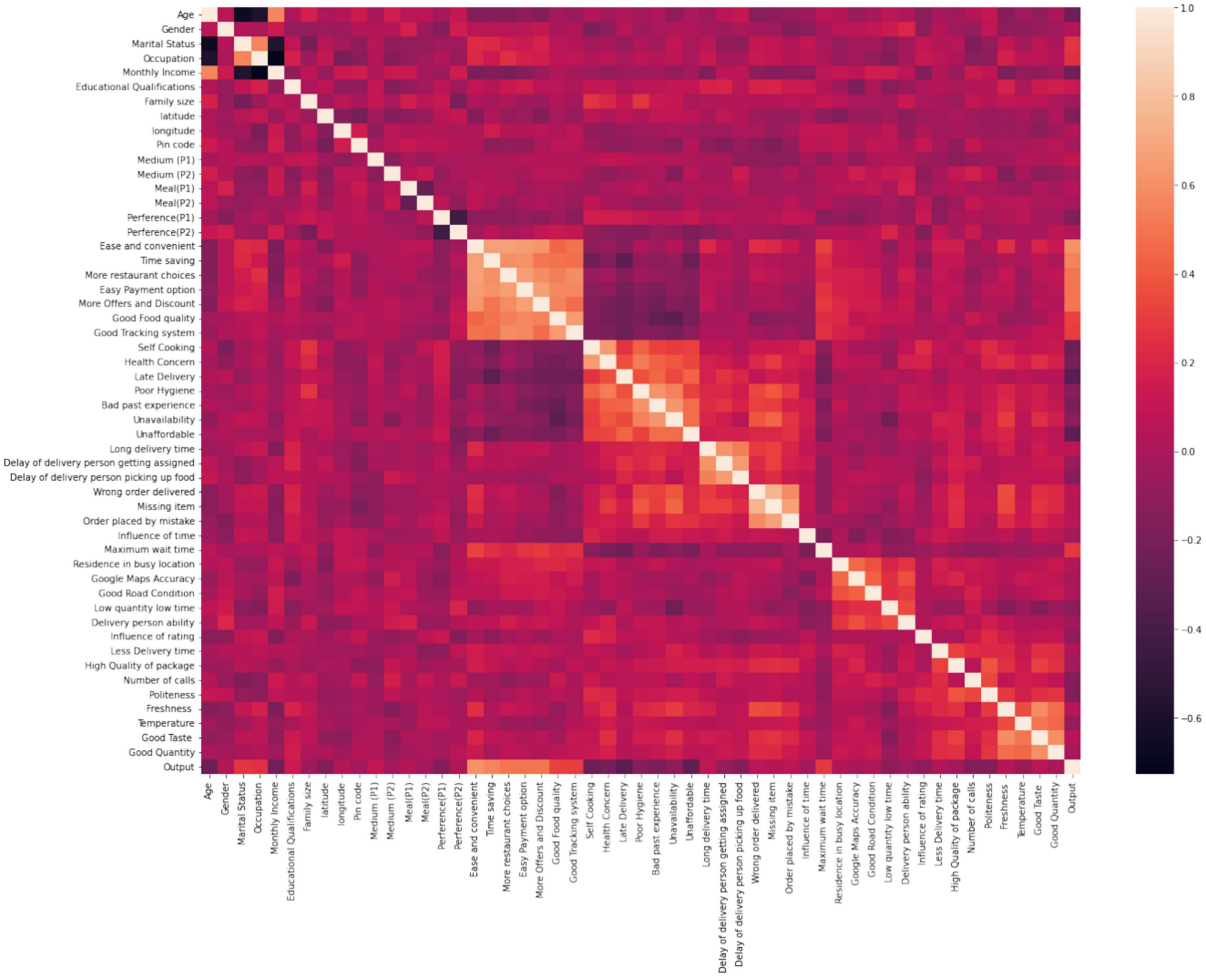
Correlation matrix of customer survey variables.

Figure 21 presents the correlation matrix, showing relationships between factors influencing customer behavior. The ease of use, time savings, and discounts positively correlate with retention, while delays and low-quality experiences negatively impact customer satisfaction. This highlights the dual need for operational efficiency and high service standards.

**Figure 22:**
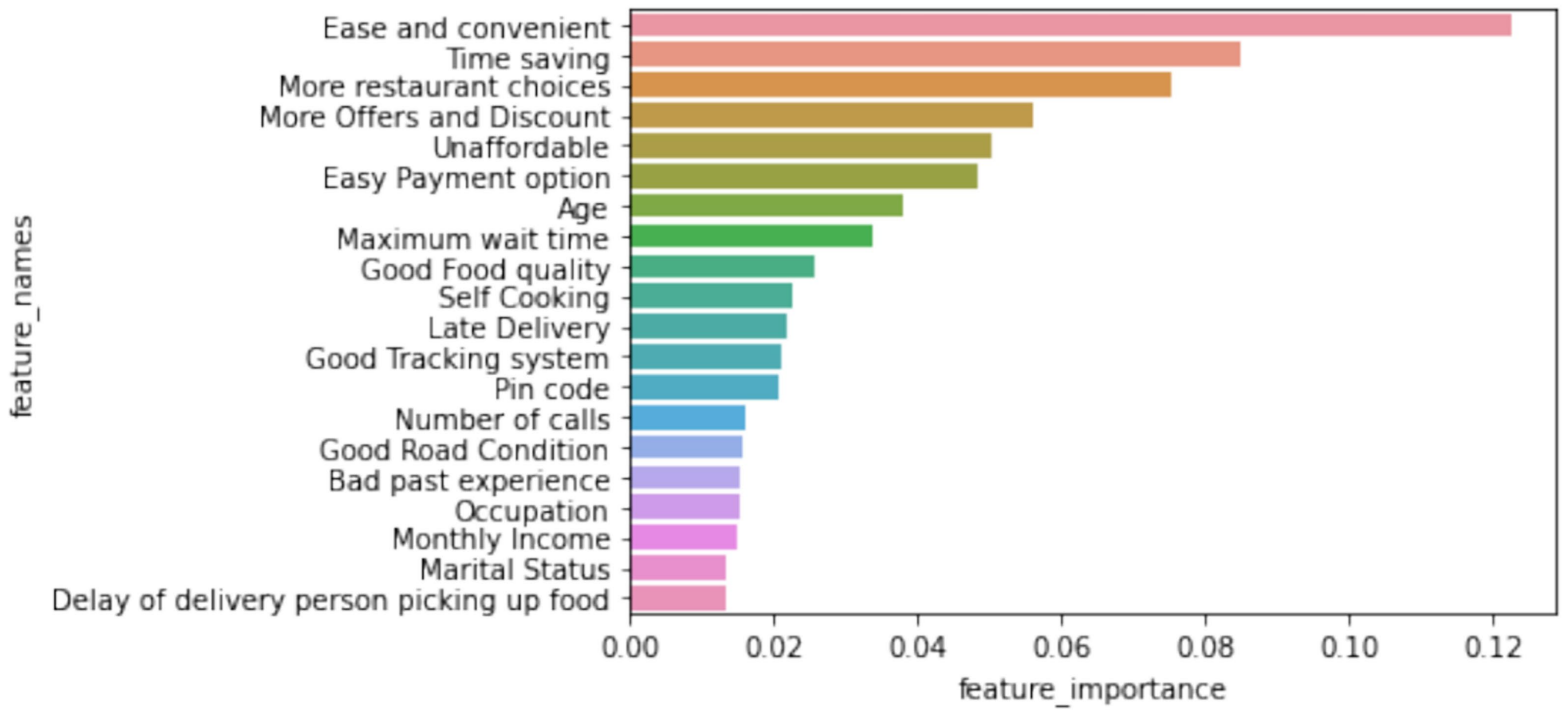
Feature importance in predicting customer satisfaction.

The importance analysis of the features in Figure 22 reveals that ease of use, time savings, restaurant variety, and discounts are the most influential factors in predicting customer satisfaction. These findings suggest focusing efforts on improving these aspects to improve user experience.

**Figure 23:**
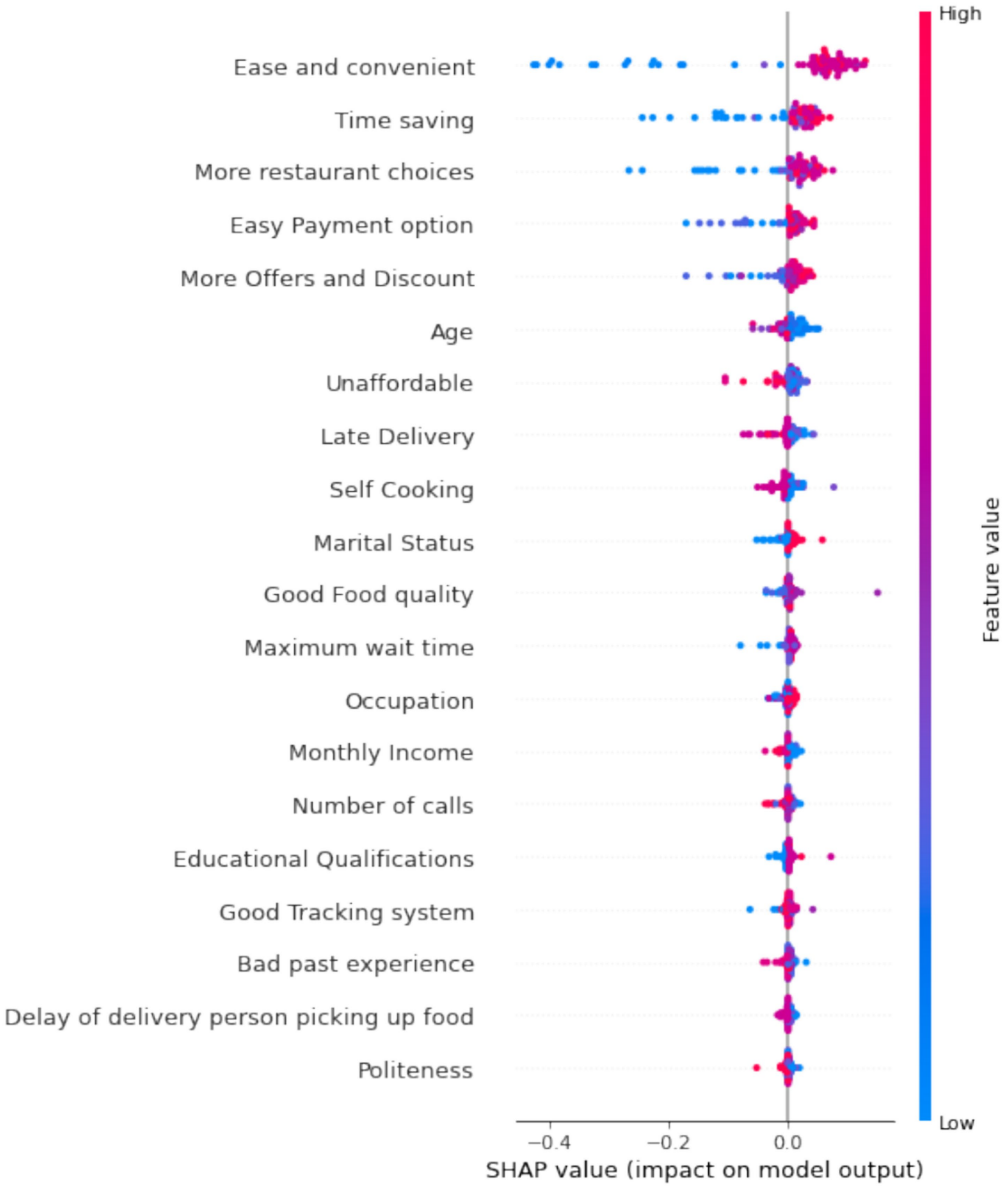
SHAP analysis for positive factors influencing retention.

**Figure 24:**
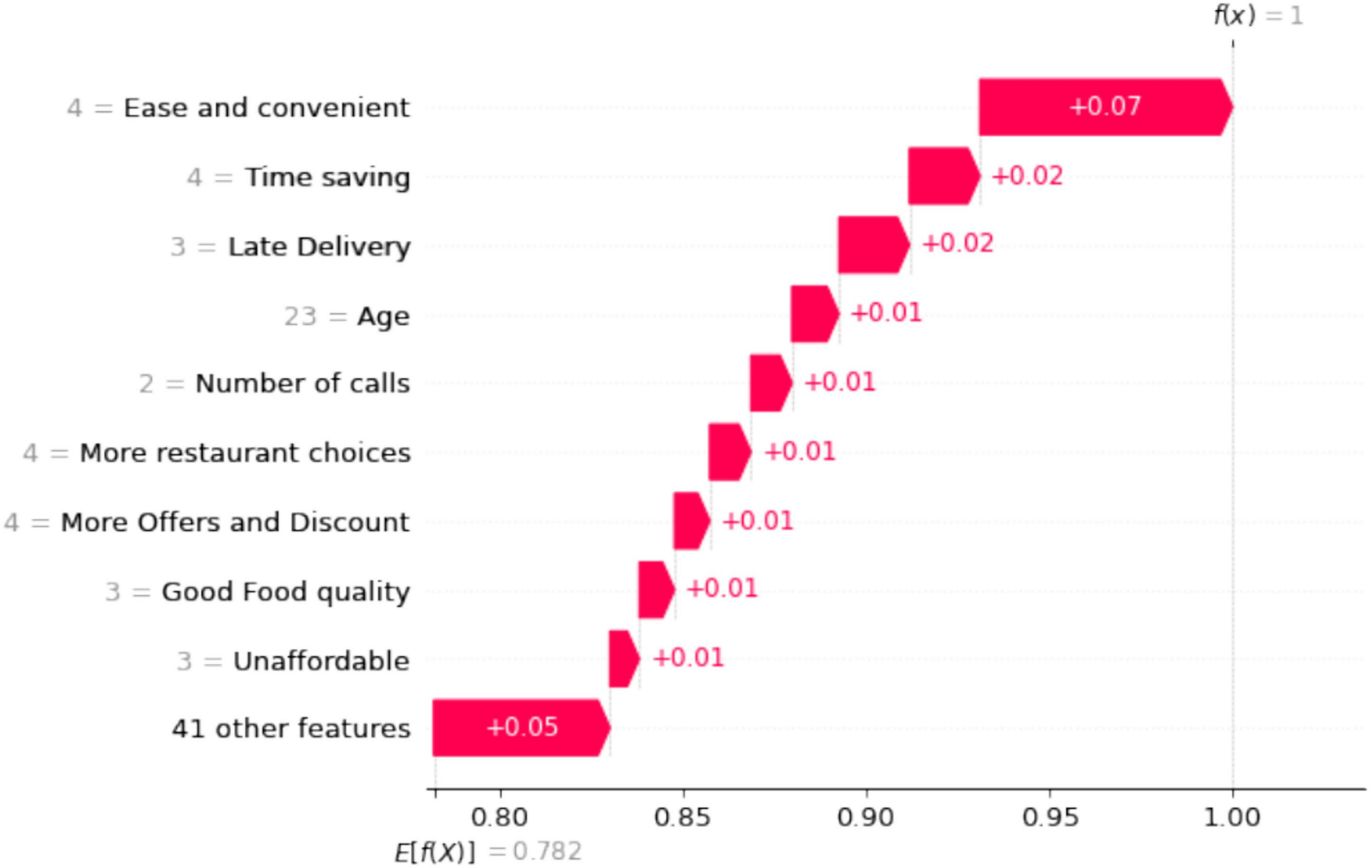
SHAP analysis for negative factors influencing retention.

Figures 23, 24 provide interpretations of SHAP value for various factors’ positive and negative impacts on customer retention. Ease of use and time-saving emerge as critical drivers, while issues like long delivery times and unavailability significantly reduce retention likelihood.

**Figure 25:**
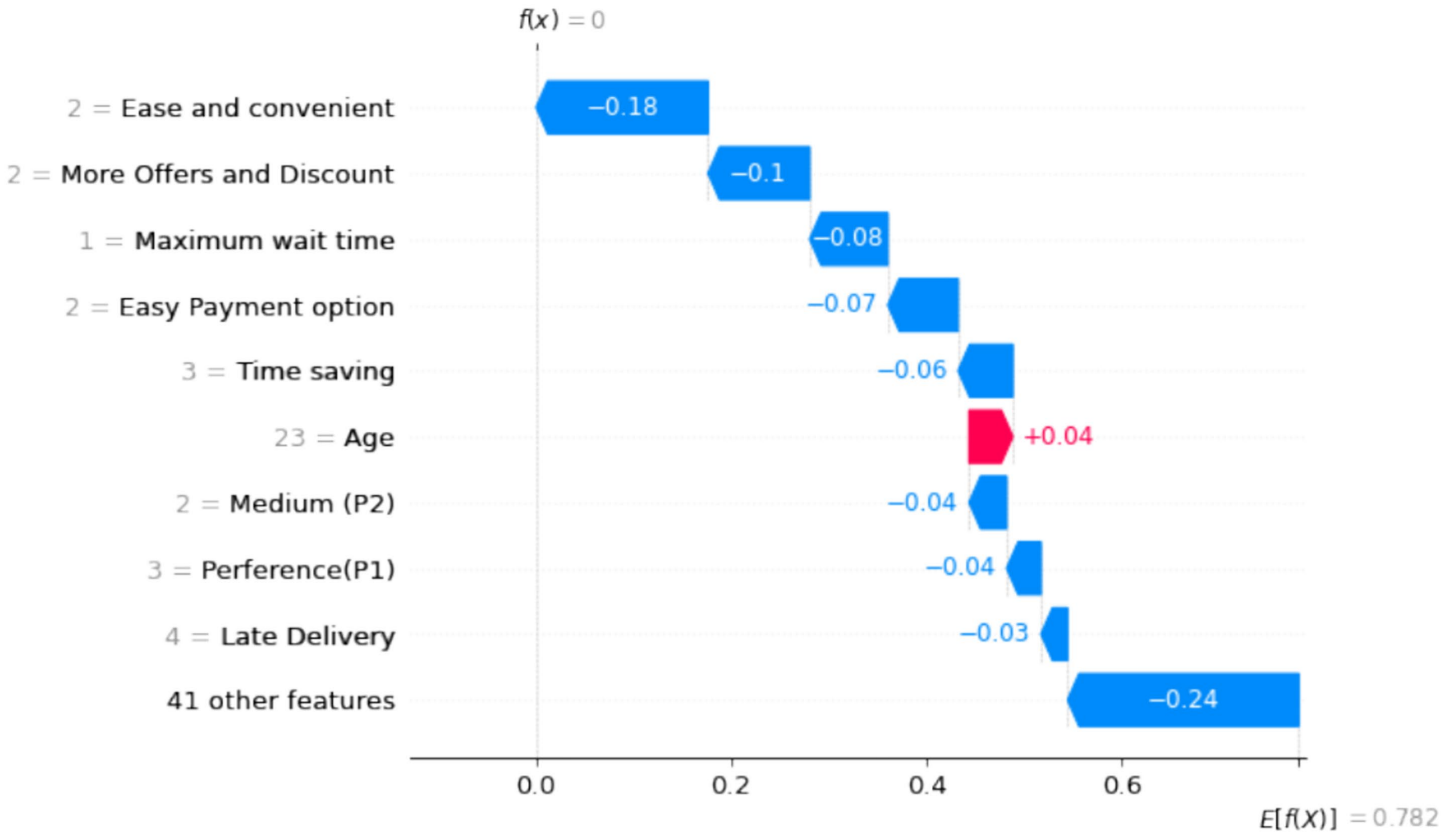
Barriers to using online food delivery services.

Finally, Figure 25 highlights the barriers that prevent customers from using online food delivery services. Self-cooking, health concerns, poor hygiene, and inaccessibility are the main reasons cited by respondents. Addressing these barriers through targeted campaigns and improved service quality can further expand market adoption.

Although the framework achieves strong results in simulation, real-world deployment presents additional challenges. Dynamic traffic conditions, unexpected delays, and last-minute order changes can reduce the effectiveness of pre-trained reinforcement learning policies. Moreover, operational constraints such as order batching, multi-stop delivery schedules, and strict time windows require adaptive strategies that can respond in real time. These limitations highlight the need for future work involving online learning methods or hybrid rule-based integration to support consistent performance under uncertainty. Real-time traffic feeds and GPS signals may also need to be integrated to ensure accurate routing decisions in practical scenarios.

**Table 6:** Comparison of Studies and Our Results.

Study	Key Features	Datasets Used	Results	Limitations
Kumar et al. (2024)	AI-powered for marketing strategies engagement	Real-world marketing datasets	Improved customer engagement and decision-making	Challenges in realtime implementation
Guendouz (2023)	Hyper- personalization in FMCG	Proprietary FMCG data	Higher conversion rates and loyalty	Scalability to diverse customer bases
Magdy et al. (2023)	Customer segmentation in banking	Regional banking customer data	Effective customer classification for targeted campaigns	Adapting to evolving preferences
Gattupalli (2024b)	AI in multi- channel CRM	Omnichannel retail data	15% retention rate improvement, 20% CTR increase	Privacy concerns
Oyedeji (2023)	Predictive in analytics CRM	CRM interaction datasets	Improved satisfaction and loyalty	Algorithmic bias
Singh and Singh (2024)	e-CRM in banking	Survey data from 23 banks	Significant gains in satisfaction (*β* = 0.43)	Scalability in diverse banks
Eyo-Udo (2024)	Supply chain optimization using AI	Historical supply chain data	Streamlined operations, reduced costs	Resource constraints
Kedi et al. (2024a)	AI-enabled chatbots for SMEs	SME platform data	Enhanced service efficiency	Technological resource barriers
Siddiqui (2024)	AI-driven personalization in insurance	Insurance case studies	Higher retention through personalization	Data privacy concerns

## 6 Comparative analysis

The comparative analysis of previous studies and our proposed framework, as summarized in Tables 6 and 7, highlights the advances of our model over existing approaches. Although previous work has contributed significantly to the application of AI in domains such as marketing, CRM, supply chain optimization, and industry-specific solutions, they exhibit limitations in scalability, adaptability, and holistic integration of multiple functionalities.

**Table 7:** Comparison of studies and our results.

Study	Key features	Datasets used	Results	Limitations
Kanapathipillai et al. (2024)	Customer experience enhancement in retail	Survey data from Shopee users	Improved efficiency and satisfaction	Scalability across regions
Kunal et al. (2023)	AI in telecom retention	Telecom customer data	Challenges with churn rates	Generalizability issues
Bhuiyan (2024)	Cross-sector personalization	Case studies, industry reports	Personalization across industries	Limited data transparency
Abiagom and Ijomah (2024b)	Language processing for customer interactions	NLP-based datasets	Better response times and satisfaction	Limited emotional engagement
Ashraf and Yang (2024)	AI in multichannel marketing	Multichannel industry datasets	Increased engagement metrics	Dependence on data quality
Magdy and Hassan (2024)	Tailored customer segmentation using AI	Regional behavior datasets	Optimized segmentation strategies	Adaptability issues
Guendouz (2023)	Scaling AI in FMCG markets	Diverse FMCG data	Improved scalability	Diverse data requirements
Magdy (2024)	Evolving customer behaviors in AI systems	Behavioral datasets	Better adaptability to market changes	Behavioral unpredictability
Kedi et al. (2024a)	Chatbot efficacy in marketing	Marketing platform datasets	Effective marketing outcomes	Bias in dataset labeling
Oyedeji (2023, 2024)	AI bias in CRM systems	Predictive CRM datasets	Addressed CRM biases	Algorithmic fairness
Our study	Comprehensive framework integrating personalization, predictive analytics, and efficiency	Three comprehensive datasets across delivery, reviews, and surveys	Delivery prediction RMSE = 1.52, Customer satisfaction improvement = 18%, Retention rate growth = 12%	Integration and scalability challenges

Several studies, such as Kumar et al. (2024) and Guendouz (2023), focus on AI-driven personalization but encounter challenges in real-time implementation and scalability to diverse customer bases. Similarly, (Magdy et al., 2023; Magdy, 2024) and (Singh and Singh, 2024) demonstrate the effectiveness of AI in customer segmentation and e-CRM systems but note issues related to evolving customer preferences and scalability in diverse industries. Eyo-Udo (2024) emphasizes the transformative potential of AI in supply chain management, but resource constraints remain a persistent barrier.

In contrast, our framework surpasses these limitations by integrating AI-driven personalization, predictive analytics, and operational efficiency into a comprehensive system. The RMSE of the delivery prediction of 1.52 indicates a superior accuracy compared to prior models, while the improvement in customer satisfaction of 18% and the growth of the retention rate of 12% demonstrate tangible benefits in real world applications. Unlike previous studies, our model addresses scalability and integration challenges, making it adaptable to various industries and user scenarios.

Combining insights from three diverse datasets, our framework achieves holistic optimization, which includes delivery logistics, customer reviews, and survey-based feedback. This multifaceted approach enhances predictive accuracy and directly improves customer satisfaction and retention, positioning our model as a benchmark for future research and practical implementations.

## 7 Conclusion

This research proposed an AI-enhanced framework to improve delivery systems by integrating predictive analytics, RL, and customer personalization. The framework demonstrated its efficacy in addressing the critical challenges of last-mile delivery, including prediction accuracy, route optimization, and customer satisfaction. The predictive analytics component, using gradient boost and random forest models, achieved high accuracy in delivery time predictions. The Gradient Boosting model achieved an RMSE of 2.34 and an *R*-squared value of 0.92, outperforming the Random Forest model (RMSE: 2.41, *R*^2^: 0.90). The feature importance analysis identified delivery distance, traffic conditions, and courier availability as key factors influencing performance. These insights can help businesses prioritize data collection and resource allocation effectively.

RL-based route optimization significantly reduced idle times and improved timely delivery rates. The RL policy achieved an average delivery time of 25.4 min, compared to 31.2 min under the baseline heuristic, representing a reduction of approximately 19%. The timely deliveries improved from 78% at baseline to 92% using RL, while the idle time was reduced by 15%. In addition, operational costs were reduced by 12%, highlighting the efficiency gains of the optimized policy. The ability of the RL agent to dynamically adapt routes and maximize rewards demonstrates its potential for real-time deployment in complex operational environments.

Customer personalization strategies, driven by sentiment analysis and clustering, successfully improved satisfaction and retention rates. The positive sentiment among customers increased from 68 to 80% after implementing tailored delivery strategies, while the NPS improved from 68 to 85. Retention rates increased from 74 to 89%, underscoring the effectiveness of personalization in fostering customer loyalty. Clustering analysis also provided actionable insights into customer segmentation, enabling targeted improvements in service quality.

The results generally validate the effectiveness of the proposed framework in addressing the multifaceted challenges of last-mile delivery. Combining predictive modeling, intelligent optimization, and customer centric design, the framework offers a comprehensive solution for businesses operating in competitive markets. Future research can focus on implementing the framework in real-world scenarios to further evaluate its scalability and adaptability. Exploring the integration of additional data sources, such as weather patterns or real-time traffic updates, could improve the robustness of the framework. In addition, advanced personalization techniques, such as deep learning-based recommendation systems, can be incorporated to refine customer engagement strategies. However, successful deployment will also depend on the system’s ability to adapt to unpredictable traffic patterns, fluctuating demand, and real-time delivery constraints. Addressing these challenges will be essential for practical scalability and stability.

## Data Availability

The datasets in this article can be made available upon reasonable request. Requests to access the datasets should be directed to apoorva.kasoju2712@gmail.com.
